# The selectivity of α‐adrenoceptor agonists for the human α1A, α1B, and α1D‐adrenoceptors

**DOI:** 10.1002/prp2.799

**Published:** 2021-08-06

**Authors:** Richard G. W. Proudman, Jillian G. Baker

**Affiliations:** ^1^ Cell Signalling Research Group Division of Physiology, Pharmacology and Neuroscience School of Life Sciences C Floor Medical School Queen’s Medical Centre University of Nottingham Nottingham UK

**Keywords:** agonist selectivity, calcium, cAMP, ERK1/2‐phosphorylation, α‐adrenoceptor

## Abstract

Highly selective drugs offer a way to minimize side‐effects. For agonist ligands, this could be through highly selective affinity or highly selective efficacy, but this requires careful measurements of intrinsic efficacy. The α1‐adrenoceptors are important clinical targets, and α1‐agonists are used to manage hypotension, sedation, attention deficit hypersensitivity disorder (ADHD), and nasal decongestion. With 100 years of drug development, there are many structurally different compounds with which to study agonist selectivity. This study examined 62 α‐agonists at the three human α1‐adrenoceptor (α1A, α1B, and α1D) stably expressed in CHO cells. Affinity was measured using whole‐cell ^3^H‐prazosin binding, while functional responses were measured for calcium mobilization, ERK1/2‐phosphorylation, and cAMP accumulation. Efficacy ratios were used to rank compounds in order of intrinsic efficacy. Adrenaline, noradrenaline, and phenylephrine were highly efficacious α1‐agonists at all three receptor subtypes. A61603 was the most selective agonist and its very high α1A‐selectivity was due to selective α1A‐affinity (>660‐fold). There was no evidence of Gq‐calcium versus ERK‐phosphorylation biased signaling at the α1A, α1B, or α1D‐adrenoceptors. There was little evidence for α1A calcium versus cAMP biased signaling, although there were suggestions of calcium versus cAMP bias the α1B‐adrenoceptor. Comparisons of the rank order of ligand intrinsic efficacy suggest little evidence for selective intrinsic efficacy between the compounds, with perhaps the exception of dobutamine which may have some α1D‐selective efficacy. There seems plenty of scope to develop affinity selective and intrinsic efficacy selective drugs for the α1‐adrenoceptors in future.

## Graphical abstract

Comparisons of the rank order of ligand intrinsic efficacy suggests little evidence for selective intrinsic efficacy between agonists for the three human α1‐adrenoceptors.

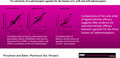

**Abbreviations**
cAMP: cyclic adenosine monophosphate
CHO: Chinese hamster ovary
HBS: Hepes buffered saline
sfm: serum free media

## 1 INTRODUCTION

Highly selective drugs are a prime goal in drug development because high‐target receptor selectivity is expected to maximize clinical effectiveness while minimizing side‐effects.^1^ For antagonist drugs, this solely involves evaluating the affinity (ability of the ligand to bind to the receptor). However, for agonists, there are two properties that need to be evaluated: affinity and efficacy (ability of the receptor‐ligand complex to induce a response).^1^, ^2^, ^3^, ^4^ A highly potent agonist could achieve this potency through high affinity or through high efficacy. Thus agonists can be highly selective due to highly selective affinity, or highly selective efficacy (where the compound could bind to several different receptors, but only activate one) or a mixture of both.^2^, ^5^

Agonist efficacy depends on several factors. Tissue and assay‐dependent factors include receptor number, receptor‐effector coupling efficiency, effector response measured, assay response window, and any desensitization that occurs within the timeframe of the measurement. This makes direct comparisons of potency (EC_50_) impossible across systems. Ligand/receptor factors are the innate ability of a certain receptor–ligand complex to induce a response and depend upon the chemical interaction between ligand and receptor. This, termed “intrinsic efficacy”, is a measure of efficacy at the molecular/single ligand–receptor level^1^, ^6^ and is a more accurate measure of true ligand efficacy than either potency or maximal response.^7^ A good way to compare the intrinsic efficacy of ligands is to remove tissue/assay factors and measure responses from individual receptor subtypes for many agonists in parallel in a null background. This allows the ligand's intrinsic efficacy to be ranked (e.g. by “efficacy ratios” *K_D_*/EC_50_ ^1^, ^3^, ^4^, ^6^) and thus compared across different receptors. Previous studies using this have found that some agonists are highly selective purely because of a highly selective binding profile, and not because of any intrinsic efficacy selectivity (e.g. salmeterol at β2‐adrenoceptors).^8^

The α1‐adrenoceptors (α1A, α1B, and α1D, Alexander et al., 2019/2020) are Gq‐coupled GPCRs expressed in a wide range of tissues including blood vessels, heart, kidney, spleen, liver, brain, and lower urinary tract.^9^, ^10^, ^11^, ^12^, ^13^, ^14^ Whereas α‐adrenoceptor antagonists (α‐blockers) are used to treat hypertension and benign prostatic hyperplasia, α‐agonists are used to manage hypotension and sedation in intensive care settings (e.g. phenylephrine,^15^ dexmedetomidine, and clonidine^16^), for ADHD (attention deficit hypersensitivity disorder e.g. guanfacine^17^, ^18^), for muscle spasm and spasticity (e.g. tizanidine^19^) but are probably most widely as over‐the‐counter nasal decongestants (e.g. oxymetazoline and xylometazoline^20^, ^21^). Thus there are many structurally different α‐adrenoceptor agonists with which to study agonist selectivity and determine how that is achieved.

In addition to α1‐adrenoceptor‐Gq‐PLC‐calcium signaling, the α1‐adrenoceptors have also been shown to stimulate other signaling cascades.^14^, ^22^, ^23^ Some recent studies have suggested that biased signaling can occur via the α1A‐adrenoceptor. Isoprenaline was thought to have α1A‐cAMP biased signaling.^24^ Oxymetazoline was initially thought to have ERK1/2‐phosphorylation bias.^25^ It was later confirmed that the “biased” responses were occurring via a different receptor although phenylephrine and methoxamine ERK1/2‐phosphorylation bias and A61603 cAMP bias were proposed.^26^ However the best way to determine whether a certain ligand is indeed an outlier inducing biased‐signaling is to examine many ligands in parallel rather than just a few.^1^

Many α1‐agonist studies examine only a few ligands, study just one receptor, or use receptors from different species, making comparing intrinsic efficacy difficult (e.g. 24, 25, 26, 27). The aim of this study was to examine the selectivity of a large range of agonists for the human α1A, α1B, and α1D‐adrenoceptors, with specific aims to identify whether agonists were selective due to selective affinity or selective intrinsic efficacy. Additionally, as several different agonist responses were examined, ligands with bias toward one signaling cascade over another would also be identified.

## 2 MATERIALS AND METHODS

### 2.1 Materials

^3^H‐prazosin, ^3^H‐adenine, Microscint 20, Ultima Gold XR scintillation fluid and the Surefire Alphascreen pERK1/2 kit were from PerkinElmer. ^14^C‐cAMP was from Hartmann Analytic. Fluo‐4AM and pluronic F‐127 were from Invitrogen. Gibco foetal bovine serum was from Fischer Scientific. All other reagents were from Sigma‐Aldrich. A list of the ligands studied with the source and supplier code is given in Table S1.

### 2.2 Ligand selection

Commercially available ligands with known α‐adrenoceptor agonist activity from the literature were investigated. In addition, several ligands generally considered to be antagonists were investigated (taken from [28]) if they were found to have agonist activity at one or more α1‐adrenoceptors. Brimonidine and UK14304 were purchased from different suppliers and as they appeared very different in solution (brimonidine was clear whereas UK14304 was bright yellow) are reported separately. Medetomidine (racemate) and its active isomer dexmedetomidine (increasingly used in intensive care units) are also reported separately.

### 2.3 Cell culture

CHO‐K1 (RIDD: CVCL_0214) stably expressing the human α1A‐adrenoceptor, human α1B‐adrenoceptor, or human α1D‐adrenoceptor (full length) were used.^28^

In addition, the parental CHO cell line without any transfected receptors was also used. Cells were grown in Dulbecco's modified Eagle's medium nutrient mix F12 (DMEM/F12) containing 10% foetal calf serum (FCS) and 2 mM L‐glutamine in a 37°C humidified 5% CO_2_: 95% air atmosphere.

### 2.4 ^3^H‐prazosin whole‐cell radioligand binding

Cells were grown to confluence in white‐sided 96‐well view plates and whole‐cell binding studies were conducted as previously described^28^ in a total well volume of 200 µl per well. Cells were incubated with ^3^H‐prazosin and competing ligand in 200 μl for 2 h in serum‐free media (sfm) at 37°C and plates counted using a Topcount (2 min per well) after a minimum of 6 h in the dark at room temperature. Total binding and non‐specific binding (tamsulosin 10 μM for α1A and α1B, and 100 µM for α1D—see^28^ for full data and explanation) were determined in every plate. ^3^H‐prazosin concentrations were determined from the average of triplicate 50 µl samples of each ^3^H‐prazosin concentration used and were in the range of 0.21 to 1.41 nM. *K_D_* values were calculated from IC_50_ values using the Cheng‐Prusoff equation (see below).

### 2.5 Intracellular free calcium mobilization

Cells were grown to confluence in black‐sided 96‐well view plates, and calcium measurements were made using a FlexStation 3 at 37°C. Cells were loaded for 45 min at 37°C with Fluo‐4AM/pluronic‐F127 in sfm containing 25 mM probenecid. Cells were washed twice with 2 × 200 μl HEPES‐buffered saline (HBS, containing 2 μM CaCl_2_). 80 µl HBS was then added to each well and the plate put into the Flexstation. Agonist ligands were diluted to five times final concentration of HBS in round bottomed 96‐well compound plates and put in the Flexstation. The Flexstation robotics added 20 µl agonist ligand from the compound plate into the existing 80 µl HBS in the cell plate (1:5 dilution). Basal and maximum responses (defined by 10 µM ionomycin) were determined in each plate. Calcium mobilization was followed for 120–200 s per well. The data were plotted as the maximum value obtained for calcium mobilization over the basal value obtained for that well before the addition of ligand.

### 2.6 ERK1/2‐phosphorylation

Extracellular signal‐regulated kinases (ERK1/2) activation was measured using a Surefire Alphascreen pERK1/2 kit as per manufacturer's instructions. Cells were grown to confluence in clear‐sided 96‐well plates, then double serum starved by washing the cells twice with 100 µl sfm before incubating in a further (third) 100 µl sfm for 24 h before experimentation. Agonists in 20 µl sfm were added to the well (contained about 80 µl after some evaporation over 24 h, thus approximately a 1:5 dilution in wells) and incubated for 2–4 min (at 37°C). Responses were initially studied at 2, 5, 10, and 15 min after addition of agonist. Responses retained a similar pattern (with regards to EC_50_ value and proportion of the of the positive control response—10 µM PDBu); however, the response window was greatest at 2 and 5 min and thus all data reported here are following 2–4 min agonist incubation. Reagents were then removed, 20 µl lysis buffer added to each well, and ERK1/2‐phosphorylation measured using the Alphascreen kit as per manufacturer's instructions. After a minimum of 2 h in the dark, the plates were read on an Envision plate reader using standard Alphascreen settings. Basal and maximum ERK1/2‐phosphorylation (as determined by 10 µM PDBu, Phorbol 12,13‐dibutyrate) was measured in each plate.

### 2.7 ^3^H‐cAMP accumulation

Cells were grown to confluence in clear‐sided 48‐well plates and ^3^H‐cAMP accumulation was measured as previously described.^5^ Following a ^3^H‐adenine load, cells were washed and incubated in sfm containing 1 mM IBMX (500 µl per well). Agonist (in 5 µl) was added and the cells were incubated for 5 h at 37°C. Basal and response to 10 µM forskolin were determined in every plate. Where used to examine Gi‐coupled responses, basal cAMP was augmented by 10 μM forskolin and inhibition of this forskolin‐induced response was examined. In these cases, forskolin was added to the wells 10 min after the addition of agonist. The assay was terminated with 50 µl concentrated HCl per well and ^3^H‐nucleotides separated by column chromatography.^5^

### 2.8 Data analysis

All pharmacological data were plotted using Graphpad Prism7.

### 2.9 Whole‐cell binding

The affinity of ^3^H‐prazosin has previously been determined from saturation binding in these cell lines.^28^ The affinity of competing ligands was determined from a one‐site sigmoidal response curve where the IC_50_ is the concentration required to inhibit 50% of the specific binding of the ^3^H‐prazosin, A is the concentration of the competing ligand and NS is the non‐specific binding (Equation 1).

(1) $$\%\;\text{uninhibited binding}=100‐\frac{\left(100\times A\right)}{\left(A+{\text{IC}}_{50}\right)}+\text{NS}.$$

The affinity (*K_D_* value) of the competing ligand was then calculated from the IC_50_ using the Cheng‐Prusoff equation (Equation 2) where [^3^H‐prazosin] is the concentration of ^3^H‐prazosin in that experiment and *K_D_* ^3^H‐prazosin is the *K_D_* value of the radioligand.

(2) $$K_{D}=\frac{{\text{IC}}_{50}}{1\;+\;\left(\left[{\;}^{3}H‐\text{prazosin}\right]/K_{D}{\;}^{3}H‐\text{prazosin}\right)}.$$

### 2.10 Functional experiments

Agonist responses were usually best described by a one‐site sigmoidal concentration response curve (Equation 3) where *E*_max_ is the maximum response, [A] is the agonist concentration and EC_50_ is the concentration of agonist that produces 50% of the maximal response

(3) $$\text{Response}=\frac{E_{\max}\times \left[A\right]}{{\text{EC}}_{50}+\left[A\right]}.$$

Some responses were best described by a two‐component response (e.g. Figure 3). Here a two‐component response curve was used (Equation 4)

(4) $$\%\text{maximum stimulation}=\frac{\left[A\right]\cdot N}{\left(\text{EC}1_{50}+\left[A\right]\right)}+\frac{\left[A\right]\cdot \left(100‐N\right)}{\left(\text{EC}2_{50}+\left[A\right]\right)},$$

where *N* is the percentage of site 1, [A] is the concentration of agonist, and EC1_50_ and EC2_50_ are the respective EC_50_ values (or IC_50_ values) for the two agonist sites. For the data in Tables 2 and 3, the log EC_50_ quote for ERK1/2‐phosphorylation is that of the initial stimulatory part of the response.

### 2.11 Efficacy ratios

Efficacy ratios were calculated by dividing the *K_D_* value by the EC_50_ value for each ligand as per method of Furchgott.^6^

(5) $$\log\;\text{efficacy}\;\text{ratio}=\frac{\log\;K_{D}}{\log{\;\text{EC}}_{50}}.$$

### 2.12 Nomenclature of targets and ligands

Key protein targets and ligands in this article are hyperlinked to corresponding entries in http://www.guidetopharmacology.org, the common portal for data from the IUPHAR/BPS Guide to PHARMACOLOGY (Harding et al., 2018), and are permanently archived in the Concise Guide to PHARMACOLOGY 2019/20.^29^

## 3 RESULTS

### 3.1 Determination of ligand affinity from ^3^H‐prazosin whole‐cell binding

**FIGURE 1:**
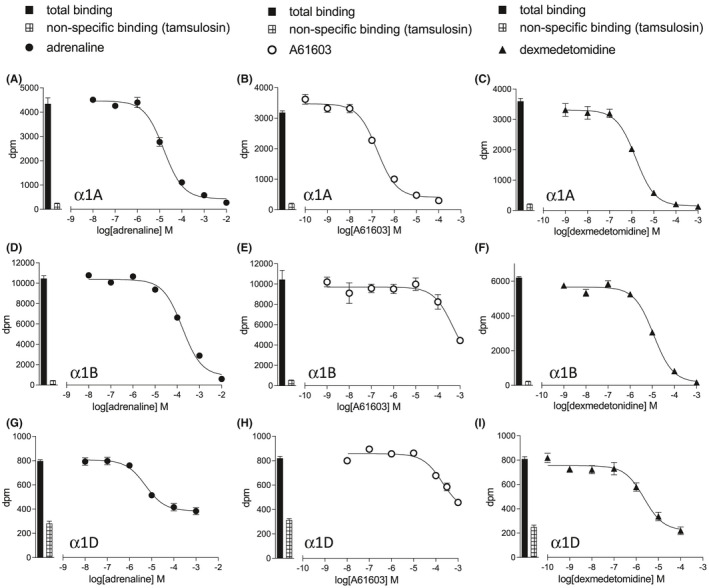
Inhibition of ^3^H‐prazosin binding to whole cells to CHO‐α1A cells (A–C), CHO‐α1B cells (D–F), or CHO‐α1D cells (G–I) by adrenaline (A, D, G), A61603 (B, E, H) or dexmedetomidine (C, F, I). Bars represent total ^3^H‐prazosin binding and non‐specific binding, determined in the presence of 10 μM tamsulosin (CHO‐α1A and CHO‐α1B) or 100 μM tamsulosin (CHO‐α1D). The concentration of ^3^H‐prazosin was (A) 0.34 nM, (B) 0.20 nM, (C) 0.22 nM, (D) 0.42 nM, (E) 0.44 nM, (F) 0.22 nM, (G) 0.58 nM, (H) 0.58 nM and (I) 0.57 nM. Data points are mean ± SEM of triplicate determinations

The affinity (*K_D_*) for ^3^H‐prazosin has previously been determined in these cell lines as 0.71, 0.87, and 1.90 nM for the α1A, α1B, and α1D‐adrenoceptor, respectively, with receptor expression levels of 1152fmol/mg protein, 4350fmol/mg protein, and 417fmol/mg protein, respectively.^28^ The α1D‐adrenoceptor is the full‐length receptor and is associated with lower levels of expression than either α1A or α1B‐adrenoceptor expression, or an N‐terminal truncated α1D‐adrenoceptor.^30^, ^31^, ^32^, ^33^ As expected therefore, the window of specific binding was smaller in the CHO‐α1D cells than the CHO‐α1A or CHO‐α1B cells (Figure 1). ^3^H‐prazosin whole‐cell binding studies yielded an affinity (log *K_D_*) for adrenaline of −5.09 in CHO‐α1A cells, −3.94 in CHO‐α1B cells, and −5.19 in CHO‐α1D cells (Table 1, Figure 1). As expected, many agonists had relatively low affinity for the α1‐adrenoceptors (Table 1, Figure 1). A61603 was the most selective agonist with an α1A‐adrenoceptor selective binding affinity of over 660‐fold (Table 1, Figure 1).

**TABLE 1:** Log K_D_ values of α‐agonists binding to the human α1A, α1B and α1D‐adrenoceptors. Values represent mean ± SEM of *n* separate experiments. Selectivity ratios are also given where a ratio of 1 demonstrates no selectivity for one receptor subtype over another. Thus, A61603 has 661‐fold higher affinity for the α1A‐adrenoceptor than the α1B‐adrenoceptor. Compounds are arranged in order of α1A‐binding selectivity

Ligand	Log K_D_ values determined from ^3^H‐prazosin whole cell binding						Affinity selectivity ratio					
	α1A	n	α1B	n	α1D	n	α1A vs α1B		α1A vs α1D		α1B vs α1D	
A61603	−6.82 ± 0.09	8	IC_50_ >−4	6	−3.92 ± 0.11	5	>661		794			>1.2
RWJ52353	−5.28 ± 0.08	5	IC_50_ >−3	5	−4.30 ± 0.12	5	>190		9.6			>20
PF3774076	−6.89 ± 0.03	5	−4.74 ± 0.04^apparent^	5	−5.24 ± 0.18	6	141		44.7			3.2
Oxymethazoline	−7.19 ± 0.07	8	−5.17 ± 0.05	6	−5.28 ± 0.04	6	105		81.3			1.3
Lisuride^a^	−7.94 ± 0.06	5	−6.07 ± 0.04	5	−6.93 ± 0.11	7	74.1		10.2			7.2
Xylometazoline	−6.94 ± 0.05	5	−5.16 ± 0.04	5	−5.23 ± 0.04	5	60.3		51.3			1.2
Dihydroergotamine	−8.62 ± 0.08	5	−6.92 ± 0.08	5	−7.19 ± 0.16^early plateau^	8	50.1		26.9			1.9
2‐MPMDQ^a^	−9.06 ± 0.07	6	−7.37 ± 0.04	6	−9.01 ± 0.16 −5.66 ± 0.29 64.0 ± 2.1% site1	8	49.0		1.1			43.7
Allyphenyline	−6.79 ± 0.04	6	−5.11 ± 0.05	7	−5.85 ± 0.05	6	47.9		8.7			5.5
Methoxamine	−4.63 ± 0.10	6	IC_50_ >−3	6	−3.82 ± 0.08	5	>42.7		6.5			>6.6
ST‐91	−5.94 ± 0.01	5	−4.39 ± 0.04	5	−5.24 ± 0.09	5	35.5		5.0			7.1
Amitraz	−5.52 ± 0.05	5	IC_50_ >−4	5	−5.08 ± 0.05	5	>33.1		2.8			>12.0
Guanfacine	−5.33 ± 0.02	6	−3.87 ± 0.06	6	−4.93 ± 0.09	5	28.8		2.5			11.5
Labetolol^a^	−7.33 ± 0.04	7	−5.91 ± 0.03	7	−6.12 ± 0.07	6	26.3		16.2			1.6
Fenoterol	−5.29 ± 0.04	5	−3.91 ± 0.10	6	−4.35 ± 0.05	5	24.0		8.7			2.8
Buspirone	−6.02 ± 0.03	6	−4.65 ± 0.03	6	−5.90 ± 0.11	6	23.4		1.3			17.8
Formoterol	−5.82 ± 0.04	5	−4.47 ± 0.04	6	−5.15 ± 0.09	5	22.4		4.7			4.8
α‐Methylnorepinephrine	−4.32 ± 0.04	5	IC_50_>−3	5	−4.84 ± 0.14^early plateau^	7	>20.9			3.3		>69.2
Atipamezole^a^	−5.99 ± 0.03	5	−4.68 ± 0.08	6	−5.33 ± 0.04	5	20.4		4.6			4.5
Isoprenaline	−4.07 ± 0.08	7	−2.80 ± 0.05^apparent^	7	−3.96 ± 0.05	5	18.6		1.3			14.5
Synephrine	−4.26 ± 0.04	5	IC_50_>−3	5	IC_50_>3.5	5	>18.2		>5.8			>3
Xylazine	−4.48 ± 0.06	5	−3.22 ± 0.05	5	−4.56 ± 0.19	6	18.2			1.2		21.9
BRL44408^a^	−5.92 ± 0.09	9	−4.68 ± 0.07	9	−5.06 ± 0.05	5	17.4			7.2		2.4
2‐PMDQ^a^	−8.19 ± 0.09	5	−6.95 ± 0.05	6	−8.42 ± 0.12 −5.61 ± 0.12 57.6 ± 2.8% site 1	9	17.4			1.7		29.5
Detomidine	−6.85 ± 0.07	7	−5.65 ± 0.02	5	−6.01 ± 0.11	7	15.8		6.9			2.3
ARC239^a^	−9.35 ± 0.08	8	−8.15 ± 0.07	9	−8.74 ± 0.12 −5.42 ± 0.21 60.5 ± 1.4% site 1	7	15.8		4.1			3.9
Eforaxan^a^	−5.47 ± 0.03	5	−4.27 ± 0.07	5	−4.97 ± 0.06	5	15.8		3.2			5.0
Adrenaline	−5.09 ± 0.07	9	−3.94 ± 0.09	10	−5.19 ± 0.14	9	14.1			1.3		17.8
CGP 12177^a^	−5.14 ± 0.05	6	IC_50_>−4	5	−4.20 ± 0.11	5	>13.8		8.7			>1.6
Sunepitrion^a^	−5.78 ± 0.06	5	−4.65 ± 0.06	5	−5.33 ± 0.23	6	13.5		2.8			4.8
Tizanidine	−5.46 ± 0.02	5	−4.35 ± 0.05	5	−5.41 ± 0.12	5	12.9		1.1			11.5
Ephedrine	−4.07 ± 0.06	5	IC_50_>−3	5	−3.57 ± 0.07^apparent^	5	>11.7		3.2			>3.7
Metaraminol	−4.07 ± 0.02	5	IC_50_>−3	5	−4.25 ± 0.09	5	>11.7			1.5		>17.8
Cirazoline	−6.17 ± 0.09	9	−5.10 ± 0.06	8	−5.53 ± 0.04	5	11.7		4.4			2.7
Moxonidine	−4.54 ± 0.03	6	−3.47 ± 0.10	7	−3.96 ± 0.05	5	11.7		3.8			3.1
para‐amino‐clonidine	−6.23 ± 0.03	6	−5.17 ± 0.06	5	−5.39 ± 0.16	6	11.5		6.9			1.7
Guanabenz	−6.48 ± 0.04	5	−5.45 ± 0.04	5	−6.02 ± 0.04	5	10.7		2.9			3.7
Noradrenaline	−4.81 ± 0.10	7	−3.79 ± 0.09	9	−5.48 ± 0.18	8	10.5			4.7		49.0
R‐phenylephrine	−4.87 ± 0.05	5	−3.87 ± 0.05	6	−4.65 ± 0.11	6	10.0		1.7			6.0
Etilefrine	−3.99 ± 0.08	6	IC_50_>−3	5	−4.45 ± 0.07	5	>9.8			2.9		>28.1
Salmeterol	−6.11 ± 0.06	5	−5.13 ± 0.04	5	−5.77 ± 0.08	6	9.5			2.2		4.4
3‐MPPI^a^	−9.57 ± 0.06	6	−8.59 ± 0.03	6	−9.76 ± 0.15 −6.93 ± 0.17 66.7 ± 3.4% site 1	7	9.5			1.5		14.8
BHT920	−4.70 ± 0.04	5	−3.73 ± 0.04	5	−4.49 ± 0.07	5	9.3		1.6			5.8
Clonidine	−6.06 ± 0.02	5	−5.13 ± 0.01	5	−5.59 ± 0.11	5	8.5			3.0		2.9
Dobutamine	−6.34 ± 0.09	8	−5.44 ± 0.04	6	−5.36 ± 0.11	6	7.9		9.5		1.2	
Salbutamol	−3.84 ± 0.06	7	IC_50_>−3	5	−3.87 ± 0.11	5	>6.9			1.1		>7.4
Dexmedetomidine	−5.88 ± 0.06	5	−5.04 ± 0.03	5	−5.91 ± 0.05	5	6.9			1.3		9.3
Naphazoline	−6.54 ± 0.05	6	−5.74 ± 0.07	7	−5.69 ± 0.12	6	6.3		7.1		1.1	
Idazoxan^a^	−5.67 ± 0.07	5	−4.88 ± 0.03	5	−5.23 ± 0.11	5	6.2		2.8			2.2
Medetomidine	−5.63 ± 0.05	5	−4.84 ± 0.03	5	−5.67 ± 0.09	5	6.2			1.1		6.8
T‐CG 1000	−5.96 ± 0.05	5	−5.18 ± 0.06	5	−5.91 ± 0.12	7	6.0		1.1			5.4
Tetrahydrozoline	−5.93 ± 0.04	6	−5.22 ± 0.06	6	−5.34 ± 0.10	5	5.1		3.9			1.3
Dopamine	−3.60 ± 0.06	7	−2.89 ± 0.09^apparent^	7	−4.09 ± 0.03	5	5.1			3.1		15.8
Brimonidine	−5.36 ± 0.04	5	−4.68 ± 0.04	5	−5.27 ± 0.06	5	4.8		1.2			3.9
UK14304	−5.53 ± 0.04	5	−4.89 ± 0.06	5	−5.36 ± 0.10	5	4.4		1.5			3.0
BHT933	−3.60 ± 0.06^apparent^	5	No binding	5	−3.70 ± 0.18^apparent^	5	>4.0			1.3	>5.0	
Rilmenidine	−4.49 ± 0.04	6	IC_50_>−4	10	−4.73 ± 0.07	5	>3.1			1.7		>5.4
Octopamine	−3.44 ± 0.07	7	IC_50_>−3	6	IC_50_>−3	5	>2.8		>2.8		1.0	
BMY7378^a^	−6.61 ± 0.05	5	−6.23 ± 0.05	6	−8.60 ± 0.13 −5.93 ± 0.37 57.7 ± 2.6% site 1	9	2.4			97.7		234
Chloroethylclonidine	−5.43 ± 0.05	5	−5.35 ± 0.07	5	−5.50 ± 0.06	5	1.2			1.2		1.4
Midodrine	IC_50_>−3	5	No binding	5	IC_50_>−3	5						
Methyldopa	No binding	5	No binding	5	No binding	5						

^apparent^the maximum concentration of competing ligand inhibited most but not all specific binding. An IC_50_ was determined by extrapolating the curve assuming that all specific binding would be inhibited if a higher concentration of competing ligand were possible. Thus an apparent K_D_ was calculated.

^early plateau^the competing ligand did not fully inhibit specific binding and the inhibition curve reached a plateau of maximal inhibition of binding. The specific binding inhibited at the α1D‐adrenoceptor was for 72.3% ± 3.8% for dihydroergotamine and 55.3% ± 5.0% for α‐methylnorepinephrine.

^a^ Data from Proudman et al., 2020. For some ligands, the binding curve obtained for inhibition of ^3^H‐prazosin specific binding at the α1D receptor was best described by a two‐component inhibition curve. Here the K_D_ value for the first component (higher affinity) and second component (lower affinity) is given with the % of the response at the first component. For further details and example graphs see Proudman et al., 2020.

### 3.2 Free intracellular calcium mobilization

**FIGURE 2:**
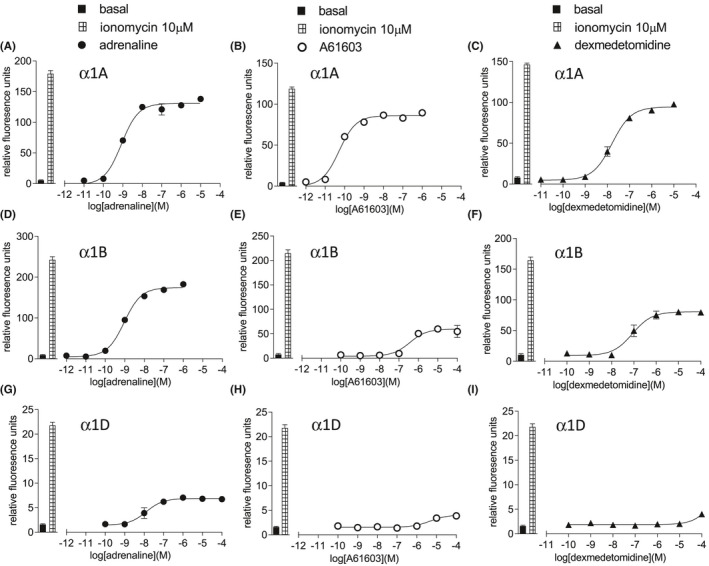
Intracellular calcium mobilization in CHO‐α1A cells (A–C), CHO‐α1B cells (D–F), or CHO‐α1D cells (G–I) in response to adrenaline (A, D, G), A61603 (B, E, H) or dexmedetomidine (C, F, I). Bars represent basal intracellular calcium release and that in response to 10 µM ionomycin alone. Data points are mean ± SEM of triplicate determinations

As all three α1‐adrenoceptors are primarily Gq‐coupled receptors, intracellular calcium mobilization was studied. In CHO‐α1A cells, adrenaline stimulated an increase in intracellular calcium (log EC_50_ = −9.09) that was 58.9% that of the response to 10 µM ionomycin (Table 2, Figure 2). This gave adrenaline an efficacy ratio of 4.00 making it the ligand with the greatest intrinsic efficacy at the α1A‐adrenoceptor (Table 2). A similar pattern was seen in CHO‐α1B and CHO‐α1D cells (Tables 3 and 4, respectively).

**TABLE 2:** Log *K_D_* values from ^3^H‐prazosin whole‐cell binding (from Table 1), log EC_50_ values, and % ionomycin maximal responses obtained from intracellular calcium mobilization and intrinsic efficacy ratios (*K_D_*/EC_50 calcium_) obtained from CHO‐α1A cells. The log EC_50_ values obtained from ERK1/2‐phosphorylation (and % PDBu responses), and those obtained from cAMP accumulation (and % forskolin maximum response), and cAMP accumulation in the presence of forskolin (with fold increase of that response) are also given

CHO‐ α1A	^3^H‐prazosin binding	Intracellular calcium release			Log efficacy ratio	ERK1/2‐phosphorylation			cAMP accumulation			cAMP accumulation (in presence of forskolin)		
	Log *K_D_*	Log EC_50_	% ionomycin	*n*	*K_D_*/EC_50_	Log EC_50_	% PDBu	*n*	Log EC_50_	% forskolin	*n*	Log EC_50_	Fold increase	*n*
Adrenaline	−5.09	−9.09 ± 0.12	58.9 ± 3.7	10	4.00	−7.74 ± 0.19^a^	71.3 ± 7.7	5	−5.63 ± 0.08	164.0 ± 7.3	5	−6.08 ± 0.16	2.65 ± 0.09	5
Noradrenaline	−4.81	−8.61 ± 0.09	62.2 ± 3.0	9	3.80	−7.49 ± 0.09^a^	84.2 ± 8.4	6	−5.46 ± 0.13	167.3 ± 13.3	5	−6.13 ± 0.13	2.73 ± 0.09	5
A61603	−6.82	−10.32 ± 0.06	61.0 ± 3.8	7	3.50	−9.92 ± 0.09^a^	74.8 ± 4.5	8	−8.05 ± 0.04	142.7 ± 8.7	5	−9.21 ± 0.07	2.19 ± 0.14	5
R‐phenylephrine	−4.87	−8.34 ± 0.09	58.3 ± 2.9	7	3.47	−7.87 ± 0.10^a^	84.5 ± 4.5	9	−5.58 ± 0.08	132.5 ± 4.5	5	−7.02 ± 0.09	2.44 ± 0.12	5
Methoxamine	−4.63	−8.06 ± 0.05	55.9 ± 2.3	8	3.43	−7.56 ± 0.14^a^	81.4 ± 4.5	6	−5.31 ± 0.05	120.0 ± 3.7	5	−6.40 ± 0.09	2.19 ± 0.09	5
α‐Methylnorepin ephrine	−4.32	−7.69 ± 0.07	65.2 ± 2.6	9	3.37	−6.86 ± 0.12^a^	87.5 ± 6.3	8	−5.09 ± 0.06	166.8 ± 7.4	5	−5.74 ± 0.10	2.49 ± 0.05	5
ST‐91	−5.94	−9.08 ± 0.11	64.6 ± 2.6	8	3.14	−8.70 ± 0.06	79.5 ± 5.2	6	−6.80 ± 0.05	108.2 ± 12.5	5	−7.86 ± 0.14	2.17 ±.10	5
Metaraminol	−4.07	−7.21 ± 0.05	66.9 ± 3.2	8	3.14	−7.01 ± 0.12	83.9 ± 4.1	6	100 μM	122.7 ± 9.8	5	−6.09 ± 0.11	2.54 ± 0.11	5
Etilefrine	−3.99	−7.11 ± 0.11	58.1 ± 2.1	9	3.12	−6.84 ± 0.05	72.8 ± 7.0	7	100 μM	82.7 ± 5.5	5	−5.89 ± 0.08	2.17 ± 0.08	5
Cirazoline	−6.17	−9.18 ± 0.11	58.2 ± 2.0	10	3.01	−9.02 ± 0.08	80.8 ± 3.6	10	−6.91 ± 0.07	108.9 ± 6.5	5	−7.97 ± 0.06	2.38 ± 0.15	5
Octopamine	−3.44	−6.10 ± 0.04	57.5 ± 2.1	8	2.66	−5.38 ± 0.14	81.2 ± 6.8	6	100 μM	18.0 ± 1.5	5	100 µM	1.89 ± 0.06	5
Dopamine	−3.60	−6.16 ± 0.06	57.9 ± 4.1	7	2.56	−5.75 ± 0.07	82.2 ± 5.0	5	1 mM	104.4 ± 7.4	5	−4.57 ± 0.15	2.75 ± 0.11	5
Para‐amino‐clonidine	−6.23	−8.70 ± 0.09	52.0 ± 2.8	10	2.47	−8.40 ± 0.10	84.5 ± 4.9	6	−6.76 ± 0.03	26.6 ± 3.2	5	−7.36 ± 0.05	1.89 ± 0.05	5
Synephrine	−4.26	−6.66 ± 0.06	55.4 ± 1.9	7	2.40	−6.22 ± 0.11	87.4 ± 8.6	7	100 μM	25.4 ± 3.4	5	−5.15 ± 0.08	2.12 ± 0.05	5
Naphazoline	−6.54	−8.93 ± 0.09	50.9 ± 2.6	7	2.39	−8.39 ± 0.07	90.9 ± 4.4	9	−6.57 ± 0.04	57.9 ± 5.7	5	−7.38 ± 0.04	2.18 ± 0.05	5
Midodrine	>−3	−5.23 ± 0.15	52.0 ± 1.7	10	>2.23	100 μM	87.7 ± 11.0	6	ND			ND		
Oxymethazoline	−7.19	−9.31 ± 0.10	54.5 ± 2.5	9	2.12	−8.97 ± 0.18^b^	92.8 ± 2.7	6	−7.18 ± 0.05	43.9 ± 5.2	5	−7.93 ± 0.13	2.33 ± 0.13	5
Medetomidine	−5.63	−7.69 ± 0.09	57.2 ± 1.8	8	2.06	−7.50 ± 0.14	85.8 ± 3.3	5	−6.19 ± 0.03	46.3 ± 5.2	5	−6.90 ± 0.06	2.12 ± 0.09	5
Clonidine	−6.06	−8.11 ± 0.07	51.6 ± 1.2	8	2.05	−7.78 ± 0.12	85.7 ± 4.7	6	−6.16 ± 0.03	33.8 ± 3.2	5	−7.15 ± 0.09	2.28 ± 0.08	5
Dobutamine	−6.34	−8.38 ± 0.11	31.3 ± 3.6	14	2.04	−7.36 ± 0.10	85.5 ± 4.3	5	−6.06 ± 0.11	11.9 ± 1.6	5	−6.18 ± 0.11	1.98 ± 0.14	5
Tizanidine	−5.46	−7.44 ± 0.09	53.5 ± 1.6	10	1.98	−7.43 ± 0.10	82.1 ± 3.9	6	−5.78 ± 0.06	65.6 ± 6.5	5	−6.91 ± 0.09	2.36 ± 0.10	5
Moxonidine	−4.54	−6.51 ± 0.18	59.3 ± 4.5	7	1.97	−6.85 ± 0.08	78.0 ± 7.2	5	ND			ND		
Guanfacine	−5.33	−7.28 ± 0.10	56.8 ± 2.2	7	1.95	−7.46 ± 0.05	87.4 ± 2.3	7	−5.54 ± 0.09	41.1 ± 2.3	5	−6.81 ± 0.13	2.22 ± 0.05	5
Dexmedetonidine	−5.88	−7.79 ± 0.10	57.3 ± 2.8	8	1.91	−7.86 ± 0.12	81.7 ± 4.4	5	−6.29 ± 0.05	57.6 ± 5.7	5	−7.07 ± 0.08	2.13 ± 0.09	5
Tetrahydrozoline	−5.93	−7.83 ± 0.12	52.4 ± 4.5	5	1.90	−7.50 ± 0.05	83.3 ± 7.1	6	−6.10 ± 0.07	34.7 ± 1.3	5	−6.79 ± 0.07	2.03 ± 0.08	5
Allyphenyline	−6.79	−8.53 ± 0.11	58.6 ± 3.0	8	1.74	−8.80 ± 0.10	79.3 ± 3.5	5	−7.05 ± 0.05	82.4 ± 5.5	5	−8.10 ± 0.08	2.38 ± 0.08	5
Xylometazoline	−6.94	−8.60 ± 0.13	64.4 ± 2.7	6	1.66	−8.42 ± 0.10^b^	98.8 ± 5.1	6	−6.90 ± 0.06	32.4 ± 5.7	5	−7.52 ± 0.13	2.00 ± 0.09	5
UK14304	−5.53	−7.17 ± 0.07	45.6 ± 1.6	7	1.64	−6.73 ± 0.11	88.7 ± 4.3	5	10 μM	12.0 ± 1.1	5	−5.86 ± 0.07	1.95 ± 0.09	5
Brimonidine	−5.36	−6.93 ± 0.10	47.8 ± 4.1	8	1.57	−6.44 ± 0.08	86.4 ± 3.6	5	−5.24 ± 0.04	17.8 ± 1.7	5	−5.80 ± 0.07	1.96 ± 0.05	5
Isoprenaline	−4.07	−5.58 ± 0.14	42.5 ± 3.7	11	1.51	−5.21 ± 0.12	72.1 ± 10.6	6	−4.09 ± 0.05	16.2 ± 3.0	5	−4.25 ± 0.04	2.22 ± 0.15	5
Xylazine	−4.48	−5.92 ± 0.13	45.1 ± 2.2	9	1.44	−5.69 ± 0.11	78.6 ± 5.8	6	ND					
Ephedrine	−4.07	−5.50 ± 0.11	43.1 ± 1.6	9	1.43	−5.12 ± 0.07	72.7 ± 6.5	7	−3.93 ± 0.09	7.8 ± 1.3	5	−4.15 ± 0.09	1.70 ± 0.10	5
Eforaxan	−5.47	−6.83 ± 0.10	45.0 ± 2.6	6	1.36	−6.12 ± 0.07	76.4 ± 5.2	6	−5.47 ± 0.10	4.0 ± 0.6	5	−5.93 ± 0.13	1.47 ± 0.13	5
BRL 44408	−5.92	−7.24 ± 0.10	36.5 ± 5.4	7	1.32	−7.03 ± 0.14	77.4 ± 4.1	6	−6.27 ± 0.17	4.7 ± 0.5	5	−6.43 ± 0.09	1.62 ± 0.08	5
Fenoterol	−5.29	−6.51 ± 0.09	30.3 ± 7.6	3	1.22	−5.69 ± 0.09	33.8 ± 6.7	6	No response		5	No response		5
Detomidine	−6.85	−8.05 ± 0.09	47.7 ± 1.8	8	1.20	−8.05 ± 0.05	84.2 ± 3.8	6	−6.85 ± 0.05	17.3 ± 1.9	5	−7.44 ± 0.06	1.95 ± 0.13	5
Rilmenidine	−4.49	−5.68 ± 0.07	45.9 ± 3.8	7	1.19	10 μM	89.0 ± 3.9	6	ND			ND		
PF3774076	−6.89	−7.94 ± 0.06	53.7 ± 4.1	5	1.05	−7.80 ± 0.11	83.9 ± 4.5	5	−7.04 ± 0.06	7.3 ± 1.4	5	−7.34 ± 0.07	1.66 ± 0.11	5
CGP 12177	−5.14	−6.18 ± 0.10	37.0 ± 2.8	5	1.04	−6.07 ± 0.12^b^	86.8 ± 2.6	5	ND			ND		
BHT920	−4.70	−5.68 ± 0.09	39.7 ± 3.3	6	0.98	−5.14 ± 0.05	61.4 ± 5.5	5	ND			ND		
Idazoxan	−5.67	−6.50 ± 0.12	25.9 ± 2.1	10	0.83	−6.21 ± 0.09	64.9 ± 2.4	6	−5.45 ± 0.07	2.6 ± 0.4	5	−5.68 ± 0.18	1.33 ± 0.06	5
Atipamezole	−5.99	−6.61 ± 0.12	44.5 ± 2.6	7	0.62	−7.00 ± 0.08	87.1 ± 5.0	5	−5.93 ± 0.01	14.5 ± 2.6	5	−6.32 ± 0.12	2.00 ± 0.10	5
Labetolol	−7.33	−7.90 ± 0.11	36.9 ± 3.7	7	0.57	−7.51 ± 0.19^b^	71.3 ± 5.2	5	−7.45 ± 0.09	4.5 ± 0.6	5	−7.39 ± 0.03	1.52 ± 0.08	5
Guanabenz	−6.48	−6.96 ± 0.13	17.8 ± 3.2	8	0.48	−6.69 ± 0.14	68.4 ± 8.6	6	−5.74 ± 0.21	2.1 ± 0.3	5	−6.84 ± 0.12	1.48 ± 0.07	5
BMY7378	−6.61	−7.04 ± 0.13	25.0 ± 3.4	8	0.43	−6.81 ± 0.13	29.0 ± 4.7	5	No response		5	100 µM	1.37 ± 0.04	5
Buspirone	−6.02	−6.43 ± 0.03	46.7 ± 3.0	8	0.41	−6.19 ± 0.08	47.3 ± 10.6	5	−5.38 ± 0.13	3.3 ± 0.06	5	−4.96 ± 0.13	1.93 ± 0.15	5
Sunepitrion	−5.78	−6.17 ± 0.14	30.1 ± 3.0	9	0.39	−5.96 ± 0.12	39.7 ± 8.4	7	ND			ND		
Lisuride	−7.94	−8.19 ± 0.11	26.4 ± 2.4	8	0.25	−7.50 ± 0.18^b^	75.2 ± 6.4	6	No response		5	No response		5
CHLOROETHYLCLONIDINE	−5.43	−5.57 ± 0.09	38.4 ± 2.8	6	0.14	−6.51 ± 0.14	66.5 ± 6.4	5	ND			ND		
2‐PMDQ	−8.19	−7.63 ± 0.13	16.6 ± 1.8	8	−0.56	−8.18 ± 0.12	10.4 ± 3.4	6	No response		5	No response		5
ARC 239	−9.35	−7.99 ± 0.19	20.8 ± 2.0	8	−1.36	−8.36 ± 0.08	29.2 ± 3.8	5	−8.41 ± 0.23	1.2 ± 0.2	5	100 µM	1.45 ± 0.06	5
2‐MPMDQ	−9.06	−7.69 ± 0.08	19.7 ± 2.0	7	−1.37	−8.27 ± 0.12	13.6 ± 3.7	5	No response		5	No response		5
Bromocryptine	−8.73	−7.31 ± 0.07	20.0 ± 3.0	6	−1.42	−7.26 ± 0.16	61.2 ± 10.3	5	No response		5	No response		5
3‐MPPI	−9.57	−7.79 ± 0.12	21.9 ± 3.6	7	−1.78	−8.25 ± 0.17	15.8 ± 4.4	6	No response		5	No response		5
RWJ52353	−5.28	10 µM	39.4 ± 2.4	5		ND			ND			ND		
Salmeterol	−6.11	10 µM	28.2 ± 2.7	6		ND			ND			ND		
BHT‐933	−3.60	100 µM	25.5 ± 3.7	5		ND			ND			ND		
T‐CG 1000	−5.96	10 µM	21.0 ± 1.9	5		ND			ND			ND		
Formoterol	−5.82	10 µM	15.0 ± 4.2	9		−6.84 ± 0.20	42.0 ± 8.0	6	No response		5	No response		5
Dihydroergotamine	−8.62	10 µM	10.0 ± 3.1	5		−8.18 ± 0.13^b^	57.2 ± 4.6	7	No response		5	No response		5
Amitraz	−5.52	No response		5		ND			ND			ND		
Salbutamol	−3.84	No response		5		100 μM	4.3 ± 1.9	5	ND			ND		
Methyldopa	No binding	No response		5		ND			ND			ND		

Note

Values represent mean ± SEM of *n* separate experiments. The ligands are arranged in order of intrinsic efficacy ratio as determined from the calcium response (EC_50_) and binding (*K_D_*).

Abbreviations: ND, not determined.

^a^ These compounds had a bi‐phasic response. Log EC_50_ and % PDBu given for initial stimulatory part of response.

^b^ These compounds stimulate ERK1/2‐phosphorylation in parent CHO cells, see Supplementary data Table S1, Figure S1: however, the responses to oxymetazoline, xylometazoline, and labetolol are more than 10‐fold more potent than the responses on the untransfected cells, so are likely to be α1‐adrenoceptor mediated.

**TABLE 3:** Log *K_D_* values from ^3^H‐prazosin whole‐cell binding (from Table 1), log EC_50_ values, and % ionomycin maximal responses obtained from intracellular calcium mobilization and intrinsic efficacy ratios (*K_D_*/EC_50 calcium_) obtained from CHO‐α1B cells. The log EC_50_ values obtained from ERK1/2‐phosphorylation (and % PDBu responses), and those obtained from cAMP accumulation (and % forskolin maximum response), and cAMP accumulation in the presence of forskolin (with fold increase of that response) are also given

CHO‐α1B	^3^H‐prazosin binding	Intracellular calcium release			Log efficacy ratio	ERK1/2‐phosphorylation			cAMP accumulation			cAMP accumulation (in presence of forskolin)		
		Log EC_50_	% ionomycin	*n*	*K_D_*/EC_50_	Log EC_50_	% PDBu	*n*	Log EC_50_	% forskolin	*n*	Log EC_50_	Fold increase	*n*
Adrenaline	−3.94	−9.41 ± 0.13	59.8 ± 3.3	9	5.47	−7.60 ± 0.15^a^	76.6 ± 9.0	6	−5.43 ± 0.02	172.1 ± 11.3	5	−5.60 ± 0.11	3.14 ± 0.09	5
Noradrenaline	−3.79	−9.23 ± 0.10	62.5 ± 1.2	8	5.44	−7.62 ± 0.04^a^	75.0 ± 8.8	6	−5.46 ±−0.02	155.5 ± 7.4	5	−5.93 ± 0.10	2.99 ± 0.06	5
R‐phenylephrine	−3.87	−9.04 ± 0.10	67.0 ± 3.3	6	5.17	−7.84 ± 0.10	77.0 ± 4.8	8	−6.11 ± 0.07	86.2 ± 5.3	5	−7.40 ± 0.11	2.43 ± 0.08	5
α‐Methylnorepin ephrine	IC_50_ > −3	−8.10 ± 0.11	69.1 ± 2.6	8	>5.10	−6.75 ± 0.13	90.6 ± 4.4	9	100 µM	129.7 ± 20.5	5	−5.56 ± 0.05	3.09 ± 0.21	5
Etilefrine	IC_50_ > −3	−7.86 ± 0.14	61.1 ± 2.1	9	>4.86	−6.52 ± 0.11	78.8 ± 6.1	7	100 µM	73.4 ± 7.1	5	−6.18 ± 0.13	2.47 ± 0.09	5
Dopamine	−2.89	−7.15 ± 0.08	62.5 ± 2.9	6	4.26	−5.83 ± 0.12	85.8 ± 6.0	7	−4.59 ± 0.08	53.0 ± 7.5	5	−5.26 ± 0.02	3.06 ± 0.14	5
Synephrine	IC_50_ > −3	−7.09 ± 0.12	57.5 ± 1.9	5	>4.09	−5.78 ± 0.08	95.1 ± 8.7	6	100 µM	23.2 ± 2.5	5	−5.94 ± 0.09	2.38 ± 0.17	5
Metaraminol	IC_50_ > −3	−6.95 ± 0.11	60.1 ± 3.1	7	>3.95	−5.84 ± 0.15	92.2 ± 3.9	6	100 µM	42.0 ± 5.1	5	−5.83 ± 0.08	2.58 ± 0.07	5
ST‐91	−4.39	−8.13 ± 0.17	62.0 ± 2.1	11	3.74	−7.33 ± 0.12	83.6 ± 8.9	6	−6.52 ± 0.04	30.4 ± 3.5	5	−7.23 ± 0.07	2.34 ± 0.09	5
Guanfacine	−3.87	−7.57 ± 0.07	59.0 ± 2.4	7	3.70	−6.71 ± 0.14	97.4 ± 2.7	7	−5.79 ± 0.04	39.0 ± 5.8	5	−6.49 ± 0.14	2.67 ± 0.18	5
Methoxamine	IC_50_ > −3	−6.64 ± 0.08	59.1 ± 3.2	7	>3.64	−5.55 ± 0.09	83.8 ± 5.7	7	100 µM	24.2 ± 4.6	5	−5.16 ± 0.12	2.42 ± 0.05	5
Isoprenaline	−2.80	−6.16 ± 0.13	53.9 ± 3.3	9	3.36	−5.36 ± 0.10	77.8 ± 8.5	7	ND			ND		
Moxonidine	−3.47	−6.49 ± 0.05	57.9 ± 2.1	7	3.02	−6.04 ± 0.15	102.0 ± 6.5	6	ND			ND		
Cirazoline	−5.10	−8.05 ± 0.10	53.3 ± 1.5	6	2.95	−6.92 ± 0.11	82.5 ± 8.6	8	−6.92 ± 0.15	11.4 ± 2.4	5	−7.40 ± 0.10	2.09 ± 0.13	5
Octopamine	IC_50_ > −3	−5.91 ± 0.13	54.9 ± 2.9	6	>2.91	−5.11 ± 0.19	76.1 ± 6.8	6	ND			ND		
Tizanidine	−4.35	−7.05 ± 0.08	48.8 ± 1.8	12	2.70	−6.54 ± 0.15	57.7 ± 5.3	6	−6.15 ± 0.17	10.7 ± 1.5	5	−6.74 ± 0.11	2.01 ± 0.11	5
Clonidine	−5.13	−7.77 ± 0.14	45.4 ± 2.1	8	2.64	−7.05 ± 0.12	60.6 ± 7.6	6	−6.60 ± 0.20	7.5 ± 0.8	4	−7.35 ± 0.10	1.67 ± 0.16	5
Para‐amino‐clonidine	−5.17	−7.79 ± 0.05	33.7 ± 2.4	8	2.62	−7.25 ± 0.08	32.8 ± 6.9	6	−7.48 ± 0.43	2.5 ± 0.6	5	−7.31 ± 0.11	1.46 ± 0.04	5
A61603	IC_50_ > −4	−6.52 ± 0.08	35.7 ± 3.7	10	>2.52	−5.75 ± 0.11	54.5 ± 7.9	9	−5.63 ± 0.18	5.3 ± 0.5	5	−6.16 ± 0.09	1.79 ± 0.05	5
Fenoterol	−3.91	−6.27 ± 0.10	38.0 ± 3.2	6	2.36	−5.56 ± 0.20	33.8 ± 7.3	7	ND			ND		
Brimonidine	−4.68	−7.01 ± 0.11	35.8 ± 2.4	8	2.33	−6.14 ± 0.08	41.2 ± 6.2	6	−6.89 ± 0.12	4.5 ± 1.6	5	−6.98 ± 0.09	1.58 ± 0.07	5
Naphazoline	−5.74	−8.03 ± 0.10	38.9 ± 2.9	8	2.29	−6.82 ± 0.09	71.1 ± 7.2	9	−7.89 ± 0.17	3.9 ± 0.7	4	−8.26 ± 0.11	1.65 ± 0.07	5
Dexmedetonidine	−5.04	−7.33 ± 0.06	43.6 ± 2.1	8	2.29	−6.88 ± 0.14	55.4 ± 7.4	6	−7.05 ± 0.15	3.7 ± 0.6	5	−7.48 ± 0.10	1.60 ± 0.07	5
UK14304	−4.89	−7.15 ± 0.05	27.5 ± 1.8	5	2.26	−6.50 ± 0.15	33.8 ± 5.0	6	−7.02 ± 0.18	2.7 ± 0.1	5	−7.01 ± 0.15	1.50 ± 0.05	5
Oxymethazoline	−5.17	−7.42 ± 0.11	27.5 ± 3.6	7	2.25	−7.33 ± 0.11^b^	67.9 ± 11.3	6	−6.66 ± 0.08	1.4 ± 0.4	4	−6.53 ± 0.12	1.20 ± 0.04	5
Salbutamol	IC_50_ > −3	−5.14 ± 0.11	58.4 ± 3.0	5	>2.14	100 µM	7.1 ± 3.5	5	ND			ND		
Xylazine	−3.22	−5.33 ± 0.14	30.4 ± 3.9	8	2.11	−5.17 ± 0.14	26.4 ± 5.8	6	ND			ND		
Medetomidine	−4.84	−6.93 ± 0.13	43.0 ± 3.4	8	2.09	−6.86 ± 0.06	55.2 ± 5.3	6	−6.94 ± 0.19	4.6 ± 0.9	5	−7.03 ± 0.06	1.52 ± 0.04	5
Formoterol	−4.47	−6.56 ± 0.14	18.6 ± 1.6	9	2.09	No response		6	No response		5	−6.55 ± 0.13	1.27 ± 0.02	5
Idazoxan	−4.88	−6.95 ± 0.12	25.7 ± 3.0	10	2.07	−6.18 ± 0.11	31.3 ± 2.2	5	−6.76 ± 0.23	2.4 ± 0.3	4	−6.84 ± 0.06	1.50 ± 0.04	5
Allyphenyline	−5.11	−7.10 ± 0.07	39.9 ± 1.9	5	1.99	−6.55 ± 0.12	65.3 ± 3.0	6	−6.76 ± 0.12	5.5 ± 0.9	5	−7.29 ± 0.10	1.87 ± 0.07	5
Detomidine	−5.65	−7.49 ± 0.11	33.8 ± 1.7	7	1.84	−7.17 ± 0.09	31.5 ± 5.4	6	−7.90 ± 0.09	2.8 ± 0.6	5	−7.89 ± 0.15	1.57 ± 0.07	5
Buspirone	−4.65	−6.42 ± 0.09	47.8 ± 3.2	5	1.77	−6.05 ± 0.18	47.2 ± 8.6	6	ND			ND		
Guanabenz	−5.45	−7.03 ± 0.12	33.7 ± 4.7	8	1.58	−5.86 ± 0.18	44.9 ± 7.9	6	−7.12 ± 0.13	2.3 ± 0.8	5	−7.09 ± 0.18	1.49 ± 0.05	5
Lisuride	−6.07	−7.23 ± 0.22	15.8 ± 2.2	12	1.16	−6.51 ± 0.16^b^	73.1 ± 8.0	5	No response		5	No response		5
Rilmenidine	IC_50_ > −3	100 µM	20.0 ± 3.9	5		No response		5	ND			ND		
Epdedrine	IC_50_ > −3	100 µM	18.5 ± 2.9	9		−4.67 ± 0.17	19.1 ± 4.0	7	ND			ND		
Atipamezole	−4.68	100 µM	18.1 ± 4.8	5		No response		5	ND			ND		
Midodrine	No binding	100 µM	16.9 ± 2.0	10		100 μM	20.4 ± 8.0	6	ND			ND		
BHT933	No binding	100 µM	16.6 ± 2.1	6		ND			ND			ND		
BHT920	−3.73	100 µM	16.3 ± 4.5	6		−5.31 ± 0.04	12.2 ± 3.6	6	ND			ND		
Salmeterol	−5.13	10 µM	15.2 ± 4.0	5		ND			ND			ND		
BRL44408	−4.68	100 µM	13.7 ± 6.5	5		100 μM	47.0 ± 7.0	6	ND			ND		
Xylometazoline	−5.16	100 μM	13.6 ± 3.3	8		−6.71 ± 0.16^b^	66.2 ± 6.0	6	No response		5	−8.04 ± 0.18^c^	13.5 ± 1.8%a	5
Chloroethylclonidine	−5.35	100 µM	10.2 ± 2.0	5		No response		6	ND			ND		
RWJ52353	IC_50_>−3	10 μM	8.1 ± 3.1	5		ND			ND			ND		
BMY7378	−6.23	100 µM	8.0 ± 2.1	9		No response		6	ND			ND		
Sunepitrion	−4.65	100 μM	8.2 ± 1.3	9		?−6.36 ± 0.29	7.8 ± 2.8	7	ND			ND		
Dihydroergotamine	−6.92	10 µM	6.4 ± 2.1	5		−7.85 ± 0.13^b^	50.1 ± 4.8	6	ND			ND		
PF3774076	−4.74	10 µM	5.2 ± 1.7	5		No response		6	ND			ND		
ARC239	−8.15	100 µM	4.8 ± 1.7	5		No response		5	ND			ND		
3‐MPPI	−8.59	100 µM	4.6 ± 1.9	7		No response		5	ND			ND		
2‐MPMDQ	−7.37	100 µM	3.1 ± 1.5	5		No response		5	ND			ND		
Dobutamine	−5.44	100 µM	3.0 ± 1.1	8		100 μM	56.3 ± 11.3	6	No response		5	−5.70 ± 0.07	1.24 ± 0.04	5
Tetrohydrozoline	−5.22	100 µM	2.9 ± 1.4	5		No response		6	ND			ND		
Labetolol	−5.91	100 µM	2.5 ± 1.2	7		−5.42 ± 0.14^b^	22.9 ± 6.3	6	No response		5	No response		5
CGP 12177	IC_50_ > −4	No response		5		−5.98 ± 0.12^b^	38.1 ± 3.8	6	ND			ND		
Eforaxan	−4.27	No response		5		100 μM	11.9 ± 2.4	7	ND			ND		
T‐CG 1000	−5.18	No response		6		ND			ND			ND		
2‐PMDQ	−6.95	No response		5		No response		7	ND			ND		
Amitraz	IC_50_ > −4	No response		5		ND			ND			ND		
Methyldopa	No binding	No response		5		ND			ND			ND		

Note

Values represent mean ± SEM of *n* separate experiments. The ligands are arranged in order of intrinsic efficacy ratio as determined from the calcium response (EC_50_) and binding (*K_D_*).

Abbreviation: ND, not determined.

^a^ These compounds had a bi‐phasic response. Log EC_50_ and % PDBu given for initial stimulatory part of response.

^b^ These compounds stimulate ERK1/2‐phosphorylation in parent CHO cells, see Supplementary data Table S1, Figure S1.

^c^ Xylometazoline caused a decrease in forskolin‐stimulated cAMP accumulation. The data given are log IC_50_ and % inhibition of forskolin‐stimulated cAMP.

**TABLE 4:** Log *K_D_* values from ^3^H‐prazosin whole‐cell binding (from Table 1), log EC_50_ values and % ionomycin maximal responses obtained from intracellular calcium mobilization and intrinsic efficacy ratios (*K_D_*/EC_50 calcium_) obtained from CHO‐α1D cells. The log EC_50_ values obtained from ERK1/2‐phosphorylation (and % PDBu responses), and those obtained from cAMP accumulation (and % forskolin maximum response), and cAMP accumulation in the presence of forskolin (with fold increase of that response) are also given

CHO‐α1D	3H‐prazosin binding	Intracellular calcium release			Log efficacy ratio	ERK1/2‐ phosphorylation			cAMP		cAMP (in presence of forskolin)		
	Log *K_D_*	Log EC_50_	% Ionomycin	*n*	*K_D_*/EC_50_	Log EC50	% PDBu	*n*	Log EC_50_	*n*	Log EC_50_	Fold increase	*n*
R‐phenylephrine	−4.65	−7.20 ± 0.10	27.8 ± 4.4	7	2.55	−6.17 ± 0.13	15.8 ± 2.9	7	No response	5	−6.77 ± 0.14	1.21 ± 0.04	5
Adrenaline	−5.31	−7.74 ± 0.10	30.3 ± 2.3	12	2.43	−6.74 ± 0.18	32.2 ± 4.0	16	No response	10	−6.06 ± 0.15	1.26 ± 0.04	10
Noradrenaline	−5.48	−7.77 ± 0.07	26.6 ± 3.6	5	2.29	−6.56 ± 0.19	30.0 ± 5.3	14	No response	5	−6.17 ± 0.15	1.20 ± 0.06	5
DOBUTAMINE	−5.36	−7.62 ± 0.15	10.6 ± 1.8	13	2.26	−6.05 ± 0.14	26.2 ± 7.5	7	No response	5	−6.51 ± 0.05	1.16 ± 0.06	5
octopamine	IC_50_ > −3	−5.20 ± 0.15	22.3 ± 4.1	9	>2.20	−4.73 ± 0.11	14.5 ± 5.9	5	No response	5	−5.74 ± 0.23	1.17 ± 0.06	5
α‐Methylnorepin ephrine	−4.84	−7.02 ± 0.10	29.9 ± 3.6	8	2.18	−5.74 ± 0.11	27.7 ± 4.9	8	No response	5	−6.05 ± 0.12	1.23 ± 0.06	5
Metaraminol	−4.25	−6.12 ± 0.13	23.1 ± 3.8	11	1.87	−5.57 ± 0.19	20.5 ± 3.0	11	No response	5	−6.49 ± 0.07	1.19 ± 0.04	5
Synephrine	IC_50_ > −3.5	−5.28 ± 0.14	16.3 ± 2.9	7	>1.78	−5.19 ± 0.14	14.5 ± 2.8	11	No response	4	No response		4
Dopamine	−4.09	−5.78 ± 0.09	23.5 ± 1.8	6	1.69	−5.13 ± 0.08	17.7 ± 4.3	7	No response	5	−5.34 ± 0.12	1.22 ± 0.08	5
Methoxamine	−3.82	−5.38 ± 0.11	22.6 ± 4.7	5	1.56	−5.08 ± 0.07	14.2 ± 3.9	6	No response	5	−4.98 ± 0.25	1.21 ± 0.04	5
Etilefrine	−4.45	−5.87 ± 0.16	13.7 ± 1.8	9	1.42	−5.82 ± 0.17	16.8 ± 2.0	9	No response	5	−6.12 ± 0.10	1.18 ± 0.04	5
A61603	−3.92	−5.29 ± 0.12	12.0 ± 3.6	8	1.37	100 μM	11.7 ± 2.9	9	No response	5	No response		5
Cirazoline	−5.53	−6.90 ± 0.09	17.3 ± 3.8	7	1.37	−5.42 ± 0.20	36.5 ± 3.2	12	No response	5	No response		5
Moxonidine	−3.96	−5.21 ± 0.11	16.6 ± 2.9	5	1.25	−4.83 ± 0.19	16.4 ± 1.6	8	No response	5	No response		5
Naphazoline	−5.69	−6.93 ± 0.12	11.8 ± 2.2	8	1.24	−4.86 ± 0.15	33.0 ± 4.7	6	No response	5	No response		5
ST‐91	−5.24	−6.34 ± 0.15	6.3 ± 1.6	8	1.10	−5.23 ± 0.21	21.1 ± 4.4	12	No response	4	No response		4
Allyphenyline	−5.85	−6.50 ± 0.21	12.3 ± 1.4	5	0.65	−5.16 ± 0.13	29.1 ± 4.7	12	No response	5	No response		5
Guanfacine	−4.93	−5.46 ± 0.15	28.6 ± 2.0	5	0.53	−5.16 ± 0.14	16.1 ± 4.0	7	No response	5	−6.64 ± 0.21	1.20 ± 0.7	5
Oxymethazoline	−5.28	−5.56 ± 0.22	7.6 ± 1.0	10	0.28	−7.26 ± 0.13^b^	48.6 ± 5.4	12	No response	5	^$^−8.24 ± 0.12	*28.7 ± 2.9%	8
Buspirone	−5.90	−5.89 ± 0.22	9.0 ± 1.5	8	−0.01	−5.59 ± 0.18	11.3 ± 2.5	10	No response	5	−5.44 ± 0.25	1.25 ± 0.06	5
Guanabenz	−6.02	100 µM	20.2 ± 5.1	6		100 μM	20.8 ± 6.6	7	No response	4	No response		4
Xylometazoline	−5.23	100 µM	18.6 ± 2.0	7		−6.92 ± 0.09^a^	70.8 ± 7.8	11	No response	5	−7.53 ± 0.22^b^	*26.5 ± 1.1%	5
Dexmedetonidine	−5.91	100 µM	13.1 ± 1.7	6		100 μM	19.7 ± 3.3	5	No response	5	No response		5
Rilmenidine	−4.73	100 µM	12.0 ± 0.9	5		No response		5	ND		ND		
Medetomidine	−5.67	100 µM	11.5 ± 2.3	7		−5.14 ± 0.20	22.6 ± 5.1	5	ND		ND		
Labetolol	−6.12	100 µM	8.1 ± 1.6	6		100 µM	36.5 ± 5.2^a^	7	ND		ND		
Clonidine	−5.59	100 µM	8.0 ± 0.5	6		100 µM	6.1 ± 1.3	7	ND		ND		
Idazoxan	−5.23	100 µM	5.7 ± 0.7	5		100 μM	2.2 ± 1.3	5	ND		ND		
BRL 44408	−5.06	100 µM	4.5 ± 2.1	6		100 µM	47.4 ± 5.7	6	ND		ND		
Fenoterol	−4.35	100 µM	4.4 ± 1.8	6		−5.40 ± 0.17	7.6 ± 1.4	9	No response	5	No response		5
BMY7378	−8.60 site 1	100 µM	4.2 ± 1.0	6		100 μM	3.0 ± 1.5	5	ND		ND		
Detomidine	−6.01	100 µM	3.8 ± 0.8	5		100 μM	12.3 ± 2.4	6	ND		ND		
Xylazine	−4.56	100 µM	3.8 ± 0.8	5		No response		5	ND		ND		
Isoprenaline	−3.96	100 µM	3.6 ± 1.2	10		−5.20 ± 0.21	8.5 ± 1.7	9	No response	5	No response		5
Eforaxan	−4.97	100 µM	2.8 ± 0.8	5		100 μM	2.7 ± 1.5	5	ND		ND		
Ephedrine	−3.57	100 µM	2.6 ± 1.3	5		100 μM	3.3 ± 2.1	5	ND		ND		
Tizanidine	−5.41	100 µM	2.5 ± 0.8	6		No response		5	ND		ND		
Sunepitrion	−5.33	100 µM	2.5 ± 0.5	5		100 μM	2.4 ± 1.1	6	ND		ND		
Tetrahydrozoline	−5.34	100 µM	2.3 ± 1.1	5		No response		5	ND		ND		
Lisuride	−6.93	10 µM	2.1 ± 0.6	5		−6.34 ± 0.11^a^	36.0 ± 5.1	6	ND		ND		
Amitraz	−5.08	No response		5		No response		5	ND		ND		
ARC 239	−8.74 site 1	No response		5		No response		5	ND		ND		
Atipamezole	−5.33	No response		5		10 μM	5.3 ± 4.6	5	ND		ND		
BHT920	−4.49	No response		5		No response		5	ND		ND		
BHT933	−3.70	No response		5		No response		5	ND		ND		
Brimonidine	−5.27	No response		6		No response		5	ND		ND		
CGP 12177	−4.20	No response		5		−5.60 ± 0.18^a^	25.8 ± 3.2	7	ND		ND		
Chloroethylclonidine	−5.50	No response		5		No response		5	ND		ND		
Dihydroergotamine	−7.19	No response		6		−8.41 ± 0.11^a^	32.9 ± 3.5	6	ND		ND		
Formoterol	−5.15	No response		5		No response		5	ND		ND		
Methyldopa	IC_50_ > −4	No response		5		No response		5	ND		ND		
Midodrine	IC_50_ > −3	No response		5		No response		5	ND		ND		
2‐MPMDQ	−9.01 site 1	No response		5		No response		5	ND		ND		
3‐MPPI	−9.76 site 1	No response		5		No response		5	ND		ND		
2‐PMDQ	−8.42 site 1	No response		5		No response		5	ND		ND		
Para‐amino‐clonidine	−5.39	No response		5		No response		5	ND		ND		
PF3774076	−5.24	No response		5		No response		5	ND		ND		
RWJ52353	−4.30	No response		6		No response		5	ND		ND		
Salbutamol	−3.87	No response		5		100 μM	4.0 ± 2.5	5	ND		ND		
Salmeterol	−5.77	No response		5		No response		5	ND		ND		
T‐CG 1000	−5.91	No response		5		10 μM	16.3 ± 5.9	6	ND		ND		
UK14304	−5.36	No response		5		No response		5	ND		ND		
Ziprasidone	−7.20	No response		5		10 μM	4.6 ± 2.7	5	ND		ND		

Note

Values represent mean ± SEM of *n* separate experiments. The ligands are arranged in order of intrinsic efficacy ratio as determined from the calcium response (EC_50_) and binding (*K_D_*).

Abbreviation: ND, not determined.

^a^ These compounds stimulate ERK1/2‐phosphorylation in parent CHO cells, see Supplementary data Table S1, Figure S1.

^b^ Oxymetazoline and xylometazoline cause a decrease in forskolin‐stimulated cAMP accumulation. The data given are log IC_50_ and % inhibition of forskolin‐stimulated cAMP as both compounds caused a decrease in cAMP accumulation.

### 3.3 ERK1/2‐phosphorylation

**FIGURE 3:**
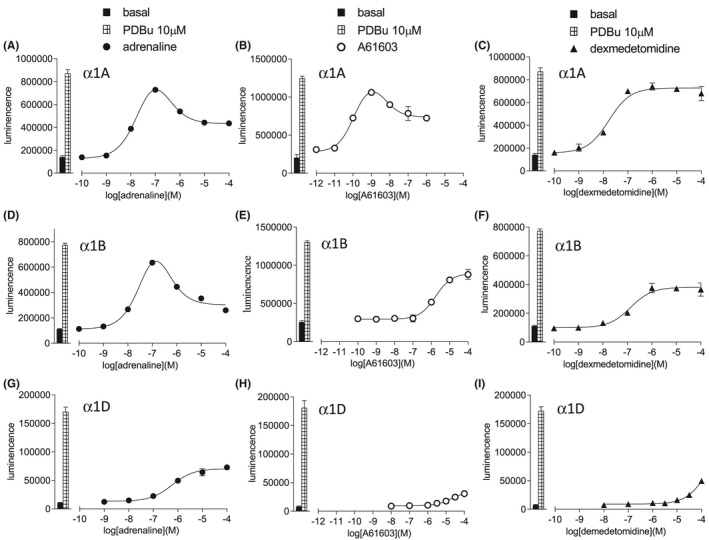
ERK1/2‐phosphorylation in CHO‐α1A cells (A–C), CHO‐α1B cells (D–F), or CHO‐α1D cells (G–I) in response to adrenaline (A, D, G), A61603 (B, E, H) or dexmedetomidine (C, F, I). Bars represent basal ERK1/2‐phosphorylation and that in response to 10 µM PDBu alone. Data points are mean ± SEM of triplicate determinations

Adrenaline stimulated an increase in ERK1/2‐phosphorylation in CHO α1A cells that was best described by a two‐component response. After an initial increase in ERK1/2‐phosphorylation (log EC_50_ −7.74, 71.3% response of 10 µM PDBu, Table 2), higher concentrations of adrenaline stimulated a lower total ERK1/2‐phosphorylation (Figure 3). This bi‐phasic dose–response pattern was seen for several of the ligands (Table 2). In CHO‐α1B cells, adrenaline stimulated a similar bi‐phasic ERK1/2‐phosphorylation response; however, only a single component response was seen in CHO‐α1D cells (Tables 3 and 4).

### 3.4 ^3^H‐cAMP accumulation

**FIGURE 4:**
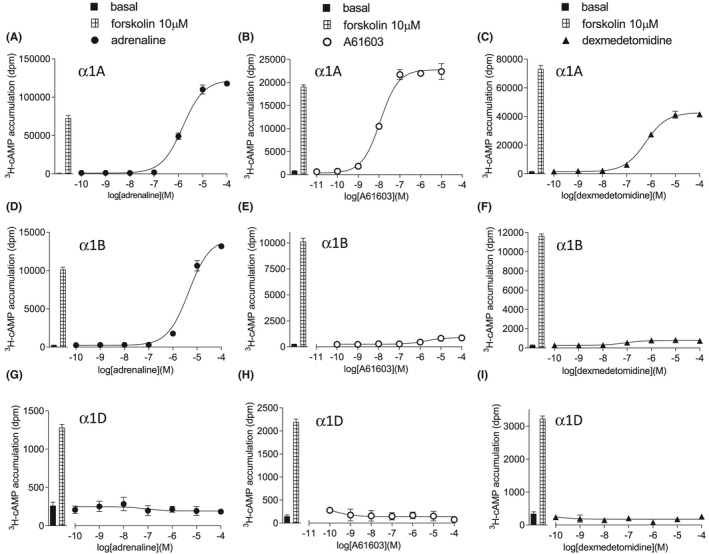
^3^H‐cAMP accumulation in CHO‐α1A cells (A–C), CHO‐α1B cells (D–F), or CHO‐α1D cells (G–I) in response to adrenaline (A, D, G), A61603 (B, E, H), or dexmedetomidine (C, F, I). Bars represent basal ^3^H‐cAMP accumulation and that in response to 10 µM forskolin alone. Data points are mean ± SEM. of triplicate determinations

**FIGURE 5:**
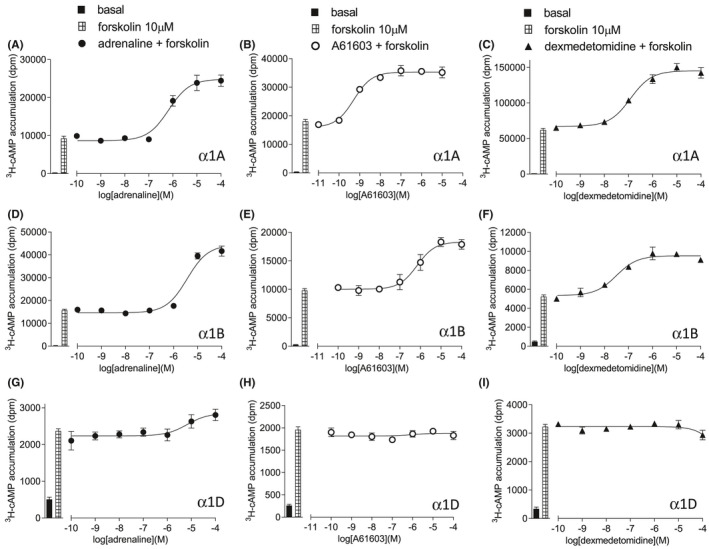
^3^H‐cAMP accumulation in the presence of 10 μM forskolin in CHO‐α1A cells (A–C), CHO‐α1B cells (D–F), or CHO‐α1D cells (G–I) induced in response to adrenaline (A, D, G), A61603 (B, E, H) or dexmedetomidine (C, F, I). Bars represent basal ^3^H‐cAMP accumulation and that in response to 10 µM forskolin alone. Data points are mean ± SEM of triplicate determinations

Adrenaline stimulated an increase in ^3^H‐cAMP accumulation in CHO‐α1A cells (log EC_50_ −5.63) that was 164% of the response seen to 10 µM forskolin (Figure 4, Table 2). This response is significantly right‐shifted when compared with the stimulatory adrenaline‐induced calcium mobilization and ERK1/2‐phosphorylation responses in these cells. To look for Gi‐mediated inhibition of cAMP, the ability of ligands to inhibit forskolin‐stimulated cAMP was examined. In CHO‐α1A cells, adrenaline did not inhibit cAMP accumulation (suggesting no Gi‐coupled response, Figure 5, Table 2). However, the stimulatory response was still seen and if anything, augmented, most likely as a result of forskolin augmentation of the Gs‐coupled response (as seen in [34, 35]). Responses were also observed in the CHO‐α1B and CHO‐α1D cells (Figures 4 and 5, Tables 3 and 4).

### 3.5 Responses in parent CHO cells without the transfected receptors

**FIGURE 6:**
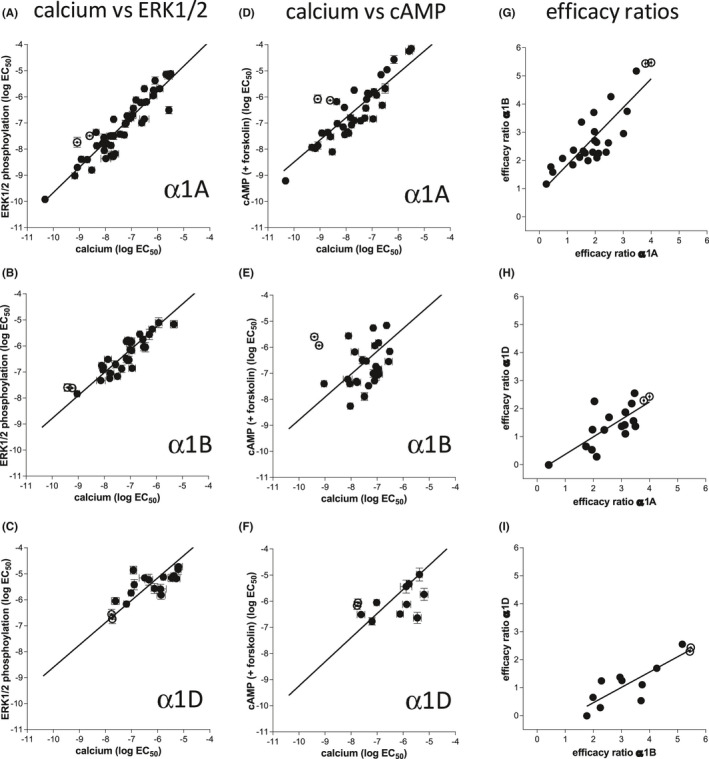
(A–C) Correlation plots of log EC_50_ determined from intracellular calcium mobilization with the EC_50_ determined from ERK1/2‐phosphorylation in (A) CHO‐α1A cells, (B) CHO‐α1B cells, and (C) CHO α1D cells. The endogenous hormones adrenaline and noradrenaline are represented by open circles. The line is that of best fit. The data for oxymetazoline, xylometazoline, dihydroergotamine, lisuride, labetalol, and CGP 12177 are not included in these plots as the compounds generated agonist ERK1/2‐phosphorylation responses in non‐transfected cells and are therefore non‐α1‐mediated responses. (D–F) Correlation plots of log EC_50_ determined from intracellular calcium mobilization with the EC_50_ determined from cAMP accumulation in the presence of forskolin in (D) CHO‐α1A cells, (E) CHO‐α1B cells, and (F) CHO‐α1D cells. The endogenous hormones adrenaline and noradrenaline are represented by open circles. The line is that of best fit. (G–I) Plots of efficacy ratio (*K_D_*/EC_50_) for (G) α1A versus α1B, (H) α1A versus α1D, and (I) α1B versus α1D as determined from whole‐cell binding affinity measurements and intracellular calcium mobilization. The endogenous hormones adrenaline and noradrenaline are represented by open circles. The line is that of best fit and the slope is not 1 and does not necessarily go through the origin as this represents a function of efficacy (i.e. differences in cell line which include receptor number, receptor‐effector coupling etc.). Compounds with the greatest perpendicular distance from the line represent compounds with the greatest degree of selective intrinsic efficacy

There were no measurable intracellular calcium mobilization dose responses in response to any of the agonists in the parent (untransfected) CHO cells (Table S1). A few compounds had a higher than basal stimulation at the highest concentration only and are given in Table S1. Oxymetazoline, xylometazoline, dihydroergotamine, lisuride, labetalol, and CGP 12177 stimulated ERK1/2‐phosphorylation responses in the parent CHO cells (Table S1, Figure S1). Oxymetazoline and xylometazoline responses had >10‐fold higher potency in the CHO‐α1A cells, suggesting these responses may be α1A‐receptor mediated. All other ERK1/2‐phosphoryulation responses to these six ligands are similar in parent cells, CHO‐α1A, CHO‐α1B,and CHO‐α1D cells and are likely non‐α1‐receptor mediated. Oxymetazoline and xylometazoline both resulted in a decrease in cAMP accumulation in the presence of forskolin in the parent cells and CHO‐α1D cells and for xylometazoline (less efficacious than oxymetazoline) in the CHO‐α1B. This cAMP inhibition was also not α1‐mediated. The stimulatory cAMP responses to oxymetazoline and xylometazoline in CHO‐α1A, and the stimulatory response to oxymetazoline in the CHO‐α1B cells are therefore likely α‐adrenoceptor mediated. These six compounds are not included in the calcium mobilization versus ERK1/2‐phosphorylation correlation plots in Figure 6A–C.

### 3.6 Correlation plots

In order to examine for any evidence of bias signaling, the log EC_50_ values for calcium mobilization were correlated with those for ERK1/2‐phosphorylation (Figure 6A–C). This suggests little evidence for biased signaling between these two responses at any of the α1‐adrenoceptor subtypes. To examine for potential calcium‐cAMP‐bias, a similar plot was constructed for calcium versus cAMP accumulation. Here, data are plotted for the augmented cAMP accumulation in the presence of forskolin as this has more ligands with measurable agonist responses and the only method by which α1D‐cAMP responses could be measured. Although this 5‐h assay has more potential for ligand degradation (especially of the catecholamines), in a similar study of β‐adrenoceptor cAMP accumulation, potency (measured at 10, 30 min, and 5 h) for both catecholamines and synthetic ligands remained the same, suggesting little loss of response due to ligand degradation.^5^ There was also little evidence for calcium versus cAMP accumulation bias at the α1A‐adrenoceptor. There was however some scatter from the line of best fit for the α1B‐adrenoceptor, suggesting some potential biased signaling. For example α‐methylnorepinephrine and naphazoline had similar calcium responses (log EC_50_ −8.10 and −8.03, respectively) but rather different cAMP accumulation responses (log EC_50_ −5.56 and −8.26, respectively).

Finally returning to a major aim of the study – to look for any evidence of intrinsic efficacy selectivity – the efficacy ratios for calcium release were compared for α1A and α1B (Figure 6g) and α1A and α1D (Figure 6h). Here, dobutamine was the ligand furthest from the line of best fit suggesting it has some α1D‐selective efficacy relative to that seen at the α1A or α1B‐adrenoceptors.

## 4 DISCUSSION

This study compared the binding affinity and functional responses of 62 compounds at the human α1A, α1B, α1D‐adrenoceptors. α1A and α1B‐adrenoceptors are present in human heart.^13^ Although α1A and α1D‐adrenoceptors are important for vasoconstriction, the role of the α1B‐adrenoceptor (also present in blood vessels) is less certain.^10^, ^14^, ^36^, ^37^ Interestingly, the affinity of adrenaline and noradrenaline was substantially lower for the α1B‐adrenoceptor than for α1A or α1D‐adrenoceptors. Adrenaline and noradrenaline had high intrinsic efficacy, with adrenaline being marginally higher at each receptor (in keeping with the slightly more potent adrenaline vs. noradrenaline responses observed by [21, 25, 38]). Phenylephrine (patented 1927, in clinical use since 1934^39^) and noted as potent by others,^25^, ^26^ also had very high intrinsic efficacy.

A61603 was the most selective agonist studied, with a calcium release and ERK1/2‐phosphorylation potency (EC_50_ value) in CHO‐α1A cells in the sub‐nanomolar range, rather than the micromolar range of the CHO‐α1B and CHO‐α1D cells. This high α1A‐potency has been previously reported.^11^, ^24^, ^25^, ^26^, ^40^ However to understand more, examination of both affinity and agonist responses is necessary. A61603 has high (>660‐fold) α1A‐adrenoceptor‐binding selectivity, and this explains its high selectivity. Other agonist compounds had α1A‐selective affinity, including PF3774076, oxymethazoline, lisuride, xylometazoline, and dihydroergotamine. No compound had α1B‐selectivity, and BMY7378 (a known α1D‐antagonist^31^, ^41^) was the only compound with α1D‐selective affinity.

Phenylephrine, naphazoline, oxymetazoline, and xylometazoline are present in many non‐prescription nasal congestion treatments. They cause α‐agonist‐induced vasoconstriction, reducing blood flow in nasal mucosa,^10^ although there is still uncertainty about their clinical value.^20^, ^42^ Even topical preparations have problems including rebound congestion (first reported by Feinberg and Friedlaender,^43^ and is still debated,^44^, ^45^) and predictable systemic α1A‐adrenoceptor complications for example hypertension and headache.^46^, ^47^ The α1A‐adrenoceptor subtype (rather than α1B or α1D), along with α2A and α2B, has the highest mRNA expression in human nasal mucosa and is thought to be the primary target.^21^ Phenylephrine (high intrinsic efficacy) and naphazoline (moderate intrinsic efficacy) were both non‐selective α1‐agonists (Tables 1, 2, 3, 4), however, both oxymetazoline and xylometazoline had α1A‐adrenoceptor selective affinity. A degree of α1A selective affinity of these two compounds has also been previously reported.^21^, ^25^, ^26^, ^38^, ^48^

Although clear agonist responses were seen with oxymetazoline for both calcium mobilization and ERK1/2‐phosphorylation in CHO‐α1A and CHO‐α1B cell lines; in CHO‐α1D cells, ERK1/2‐phosphorylation responses were substantially greater than the α1D‐calcium response raising the possibility of α1D‐biased‐signaling. Examination of other ligands reveals several compounds with substantial ERK1/2‐phosphorylation relative to calcium responses in the CHO‐α1D cells (e.g. oxymetazoline, xylometazoline, dihydroergotamine, and lisuride). Studies in untransfected parent CHO cells revealed similar agonist responses (see Supplementary data). Thus the ERK1/2‐phosphorylation responses in CHO‐α1D cells (low receptor expression) were not occurring via the transfected receptor and were not due to biased signaling. Indeed, with the exception of oxymetazoline and xylometazoline in CHOα1A‐cells where the responses were more potent, these agonist responses measured in any of the cell lines are unlikely to be α1‐adrenoceptor mediated.

An “impossible” situation of negative efficacy ratios was seen for 2‐PMDQ, ARC239, 2‐MPMDQ, and 3‐MPPI in CHO‐α1A cells: a higher concentration was required to stimulate agonist responses (EC_50_) than required to occupy the receptors (*K_D_*). These compounds had the smallest responses when compared with the ionomycin control. No agonist responses were observed in parent CHO cells, nor in CHO‐α1B or CHO‐α1D cells, suggesting that they are indeed α1A‐adrenoceptor‐mediated responses. A similar “impossible” situation occurs in β1 and β3‐adrenoceptors, where certain lower efficacy compounds activate a secondary agonist conformation^49^, ^50^, ^51^ involving the extracellular end of transmembrane 4.^52^ A “low” affinity state of the α1A‐adrenoceptor has been previously proposed (α1L), initially reported as having a lower prazosin affinity^10^ and references therein) but also seen with affinity measurements in functional assays.^11^ Further studies are required to determine whether the low potency of these agonists are occurring at a lower affinity α1A‐secondary agonist conformation, akin to that of the α1and α2‐adrenoceptors, and whether this has any relationship of this to the “α1L”‐adrenoceptor.

Overall, there was very close alignment between the calcium mobilization and the ERK1/2‐phosphorylation responses (Figure 6a–c), suggesting no biased Gq/calcium versus ERK1/2‐signaling in CHO‐α1A, CHO‐α1B, or CHO‐α1D cells. Copik et al.^24^ examined α1A‐adrenoceptor isoprenaline responses in HEK cells in detail and concluded that although isoprenaline induced similar calcium and ERK1/2‐phosphorylation responses, isoprenaline did not induce phospholipase C or inositol phosphate responses. They concluded that their calcium response was a non‐Gq‐coupled event, and thus isoprenaline was an ERK versus Gq‐biased ligand. Evans et al.^25^ and da Silva et al.^26^ report phenylephrine and methoxamine as having ERK versus Gq‐calcium bias. In our study, phenylephrine, methoxamine, and isoprenaline have different intrinsic efficacies, but no calcium versus ERK‐phosphorylation bias. It is possible that the ERK1/2‐phosphorylation in our study could be downstream from the calcium response (as suggested by [22]).

Previous studies suggest that α1‐adrenoceptors stimulate cAMP.^24^, ^38^, ^53^, ^54^ CHO‐α1A and CHO‐α1B agonist cAMP responses were seen with several compounds, although not in the α1D cells. In CHO‐α1A cells, cAMP responses required much higher agonist concentrations than that required for calcium release (as in [24, 25, 26, 54]). This lower potency Gs‐coupling is similar to that seen at the adenosine A1 receptor^55^ and may represent a lower agonist affinity for the Gs‐coupled conformation of the α1‐adrenoceptors than for the Gq‐coupled conformation. This was not always the case for α1B‐ see below.

There was no inhibition of forskolin‐stimulated cAMP in CHO‐α1A or CHO‐α1B cells, suggesting no evidence for Gi receptor coupling. In fact, forskolin further increased the cAMP stimulatory responses, in keeping with forskolin‐induced enhancement of GPCR‐Gs‐adenylyl cyclase coupling (proposed by [35] and [34]), and da Silva et al.^26^ who were not able to measure a oxymetazoline‐cAMP response, but observed an oxymetazloline response in the presence of 1 μM forskolin. In CHO‐α1D cells, an inhibitory cAMP response was seen with oxymetazoline and xylometazoline, similar to that seen in parent CHO cells, suggesting that this was not α1D‐receptor mediated. Thus oxymetazoline and xylometazoline cause non‐α1‐adrenoceptor‐mediated responses in CHO cells that decrease cAMP and stimulate significant ERK‐phosphorylation, very much in keeping with the CHO Gi‐coupled 5HT‐1B receptor proposed by da Silva et al.^26^ The stimulatory response seen in CHO‐α1A and CHO‐α1B cells is likely receptor‐mediated due to the higher level of transfected α‐adrenoceptors in these cell lines.

There was a good correlation between calcium mobilization and cAMP stimulation in CHO‐α1A‐cells suggesting little calcium versus cAMP biased signaling. However, the correlation plot for the α1B‐adrenoceptor shows substantially more scatter with adrenaline, noradrenaline, and α‐methylnorepinephrine having substantially more potent calcium than cAMP responses, whereas naphazoline, dexmedetomidine, medetomidine, allyphenyline, detmonidine, guanabenz, and dobutamine had more potent cAMP responses than calcium. There may therefore be some bias signaling with respect to calcium and cAMP pathways via the α1B‐adrenoceptor.

In α1A‐cells, six ligands stimulated biphasic ERK1/2‐phosphorylation responses: an initial increase in phospho‐ERK1/2‐phosphorylation was followed by a decrease at higher agonist concentrations (Figure 3). This appears to be an efficacy driven phenomena because these six ligands had the highest intrinsic efficacy as determined from the calcium release assay. This phenomena was also seen with adrenaline and noradrenaline in CHO‐α1B cells, but not in CHO‐α1D cells (lower receptor expression) where all responses were smaller relative to the PDBu response. Interestingly,^22^ proposed that α1A‐induced cAMP stimulation could have a negative effect on ERK1/2‐phosphorylation. Thus the Gs‐coupled cAMP stimulation, which only occurs at higher agonist concentrations, could be the explanation for the decrease in ERK1/2‐phosphorylation seen at higher agonist concentrations.

Finally, the intrinsic efficacy of ligands was examined. Although direct EC_50_ comparisons are not possible across cell lines, the rank order of intrinsic efficacies are either as presented in Tables 2, 3, 4 or pictorially from correlation plots (Figure 6). There was a good correlation for the intrinsic efficacy of agonists at these receptors, suggesting little intrinsic activity selectivity. The ligand with the most selective intrinsic efficacy was dobutamine (ranked 4^th^ in the α1D table, and furthest from the line of best fit, Figure 6). Dobutamine stimulated a response with similar affinity, potency, and intrinsic efficacy to that of noradrenaline in the CHO‐α1D cells, but despite a similar affinity, did not stimulate any measurable calcium or ERK1/2‐phosphorylation CHO‐α1B response and only a mid‐table response intrinsic efficacy response in the CHO‐α1A cells. Dobutamine has previously been shown to have affinity for α1‐adrenoceptors.^56^ However, A61603 apart, given the lack of selectivity of most α1‐adrenoceptor agonists, there seems plenty of scope to develop both affinity‐selective and intrinsic efficacy‐selective agonist drugs for the α1‐adrenoceptors in future.

## DISCLOSURE

JGB is on the Scientific Advisory Board for CuraSen Therapeutics. The majority of the data in this study predates that appointment.

## AUTHOR CONTRIBUTIONS

JGB designed the research study. JGB and RGWP performed the research. JGB analyzed the data. JGB wrote the paper.

## Supporting information

Fig S1‐S6

Table S1

## Data Availability

Data available on request from the authors: The data that support the findings of this study are available from the corresponding author upon reasonable request. Some data may not be made available because of privacy or ethical restrictions.
