# Supplementary material for: The selectivity of α‐adrenoceptor agonists for the human α1A, α1B, and α1D‐adrenoceptors
Source: Pharmacol Res Perspect. 2021 Aug 6;9(4):e00799. doi: 10.1002/prp2.799 (PMC8343220; doi:10.1002/prp2.799)
Supplement: Supplementary file 1 — Fig S1‐S6 [file PRP2-9-e00799-s002.pptx]

## Slide 1
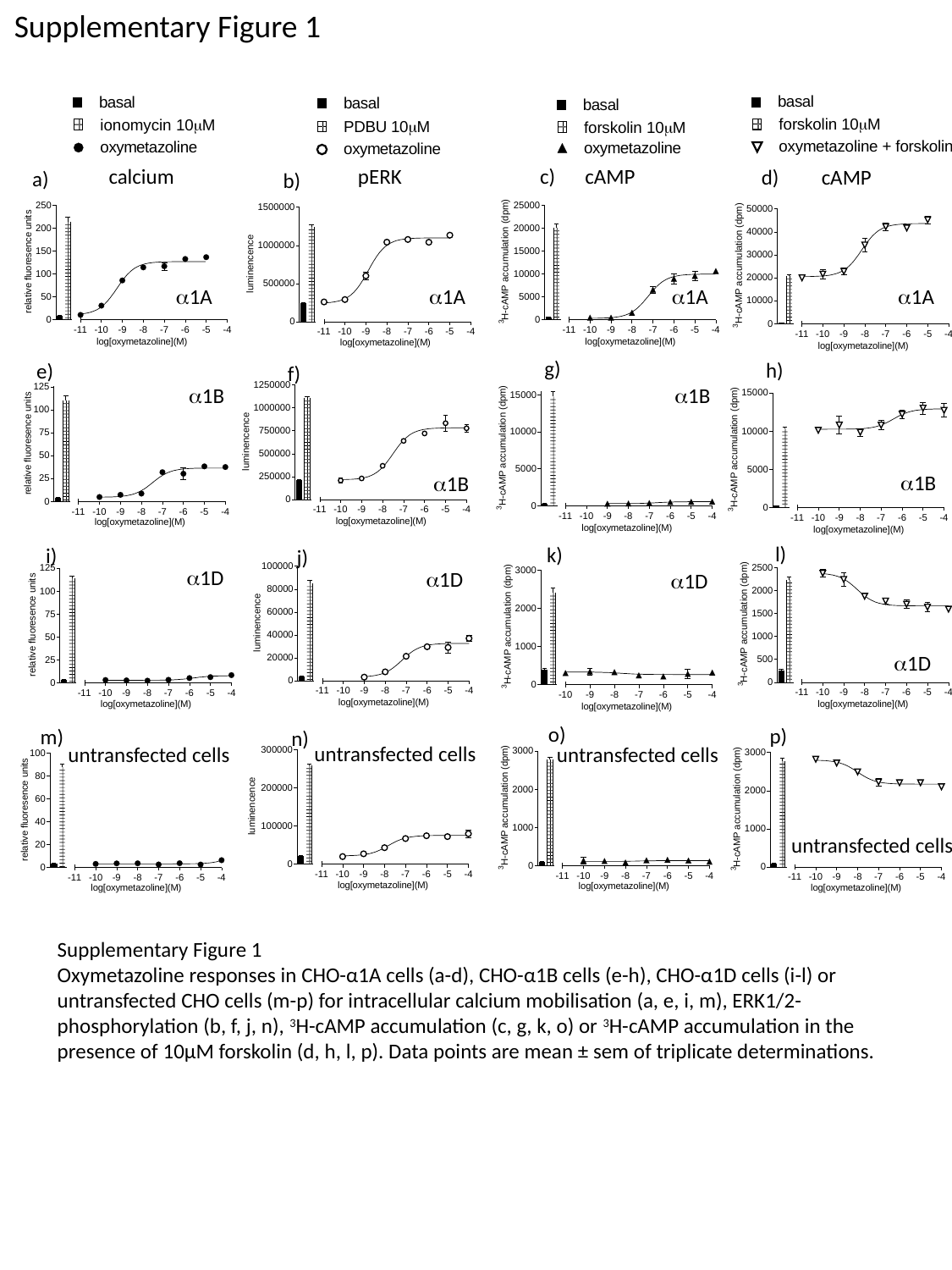

Supplementary Figure 1
c)
cAMP
pERK
calcium
d)
cAMP
a)
b)
a1A
a1A
a1A
a1A
g)
h)
e)
f)
a1B
a1B
a1B
a1B
l)
k)
i)
j)
a1D
a1D
a1D
a1D
o)
p)
m)
n)
untransfected cells
untransfected cells
untransfected cells
untransfected cells
Supplementary Figure 1
Oxymetazoline responses in CHO-α1A cells (a-d), CHO-α1B cells (e-h), CHO-α1D cells (i-l) or untransfected CHO cells (m-p) for intracellular calcium mobilisation (a, e, i, m), ERK1/2-phosphorylation (b, f, j, n), 3H-cAMP accumulation (c, g, k, o) or 3H-cAMP accumulation in the presence of 10μM forskolin (d, h, l, p). Data points are mean ± sem of triplicate determinations.
