# Supplementary material for: The selectivity of α‐adrenoceptor agonists for the human α1A, α1B, and α1D‐adrenoceptors
Source: Pharmacol Res Perspect. 2021 Aug 6;9(4):e00799. doi: 10.1002/prp2.799 (PMC8343220; doi:10.1002/prp2.799)
Supplement: Supplementary file 2 — Table S1 [file PRP2-9-e00799-s001.docx]

**The selectivity of α-adrenoceptor agonists for the human α1A, α1B and α1D-adrenoceptors**

Running title: α1A, α1B, α1D-adrenoceptor agonist selectivity

Richard G.W. Proudman, Jillian G. Baker

Cell Signalling Research Group,

Division of Physiology, Pharmacology and Neuroscience,

School of Life Sciences,

C Floor Medical School,

Queen’s Medical Centre,

University of Nottingham,

Nottingham,

NG7 2UH,

UK.

Address correspondence to: Prof Jillian G Baker

Tel: 0115 8230085

Email: [jillian.baker@nottingham.ac.uk](mailto:jillian.baker@nottingham.ac.uk)

ORCID ID: 0000-0003-2371-8202

Supplementary Table 1

Ligands (arranged in alphabetical order), with supplier and catalogue number and maximum concentration used is functional assays. The response in CHO cells without any transfected receptors are given for intracellular calcium mobilisation, ERK1/2-phosphorylation, cAMP accumulation and forskolin-stimulated cAMP accumulation. Some compounds only stimulated a response at maximum concentration only. An EC_50_ value was therefore not obtainable and % control values at the maximum concentration used are given. Values represent mean ± s.e.m. of n separate experiments. Ionomycin (10μM) stimulated a response 27.2 ± 1.2 fold over basal (n=6), PDBu (10μM ) stimulated a response 7.5 ± 0.9 (n=7) and forskolin (10μM) stimulated a response 39.5 ± 3.2 (n=6) fold over basal.

| CHO | Supplier and  catalogue number | Maximum  concentration | Intracellular calcium  release | | ERK1/2-phosphorylation | | | cAMP accumulation | | cAMP accumulation  (in presence of forskolin) | | |
| --- | --- | --- | --- | --- | --- | --- | --- | --- | --- | --- | --- | --- |
|  |  |  | % ionomycin | n | Log EC_50_ | % PDBu | n | % forskolin | n | Log EC_50_ | % forskolin-  stim cAMP | n |
| A61603 | Tocris – 1052 | 100μM | No response | 4 | No response | | 4 | No response | 5 | No response | | 5 |
| adrenaline | Sigma – E4642 | 100μM | No response | 5 | No response | | 5 | No response | 10 | No response | | 10 |
| allyphenyline | Sigma – SML1484 | 100μM | 5.9 ± 1.0 | 3 | 100μM | 12.7 ± 3.1 | 5 | No response | 5 | No response | | 5 |
| amitraz | Sigma – 45323 | 10μM | No response | 3 | No response | | 4 | ND | | ND | | |
| ARC 239 | Sigma A5736 | 10μM | 3.8 ± 2.5 | 3 | No response | | 4 | No response | 5 | No response | | 5 |
| atipamezole | Sigma – A9611 | 10μM | No response | 3 | 10μM | 3.1 ± 1.9 | 6 | No response | 5 | No response | | 5 |
| BHT920 | Tocris - 2759 | 100μM | No response | 3 | 100μM | 3.8 ± 1.8 | 6 | ND | | ND | | |
| BHT-933 | Tocris -2758 | 100μM | 1.4 ± 0.8 | 3 | No response | | 5 | ND | | ND | | |
| BMY7378 | Sellakchem S2691 | 100μM | 7.8 ± 2.2 | 3 | No response | | 5 | No response | 5 | No response | | 5 |
| brimonidine | ARK - AK35795 | 100μM | No response | 3 | 100μM | 2.3 ± 1.7 | 5 | No response | 5 | No response | | 5 |
| BRL 44408 | Sigma – B4559 | 100μM | No response | 3 | 100μM | 17.6 ± 8.1 | 5 | No response | 5 | 100μM | 77.6 ± 2.3 | 5 |
| buspirone | Sigma – B7148 | 100μM | 3.1 ± 2.3 | 3 | No response | | 5 | No response | 5 | 100μM | 113.6 ± 5.2 | 5 |
| CGP 12177 | Sigma – C125 | 100μM | No response | 3 | -6.01 ± 0.41 | 22.8 ± 4.3 | 6 | ND | | ND | | |
| chloroethylclonidine | Sigma – B003 | 100μM | 3.3 ± 3.3 | 3 | No response | | 4 | ND | | ND | | |
| cirazoline | Sigma – C223 | 100μM | 4.5 ± 0.8 | 3 | 100μM | 33.4 ± 7.6 | 7 | No response | 5 | 100μM | 82.4 ± 2.8 | 5 |
| clonidine | Sigma – C7897 | 100μM | 1.6 ± 1.0 | 3 | No response | | 6 | No response | 5 | No response | | 5 |
| detomidine | Sigma - 34265 | 100μM | 4.3 ± 1.7 | 3 | 100μM | 6.1 ± 4.0 | 7 | No response | 5 | 100μM | 83.2 ± 4.9 | 5 |
| dexmedetomidine | Sigma – SML0956 | 100μM | 6.3 ± 4.8 | 3 | 100μM | 5.8 ± 2.2 | 7 | No response | 5 | 100μM | 84.4 ± 4.7 | 5 |
| dihydroergotamine | Tocris - 0457 | 10μM | No response | 3 | -8.65 ± 0.06 | 35.9 ± 4.2 | 5 | No response | 5 | 10μM | 77.4 ± 1.9 | 5 |
| dobutamine | Sigma – D0676 | 100μM | No response | 4 | 10μM | 16.4 ± 5.9 | 6 | No response | 5 | No response | | 5 |
| dopamine | Sigma – H8502 | 100μM | No response | 3 | No response | | 4 | No response | 5 | No response | | 5 |
| eforaxan | Tocris - 0792 | 100μM | No response | 3 | No response | | 4 | No response | 5 | No response | | 5 |
| ephedrine | Sigma - 285749 | 1mM | 5.3 ± 1.6 | 3 | No response | | 5 | No response | 5 | No response | | 5 |
| etilefrine | ARK – AK390 | 100μM | No response | 4 | No response | | 4 | No response | 5 | No response | | 5 |
| fenoterol | Sigma – F1016 | 100μM | No response | 3 | No response | | 5 | No response | 5 | No response | | 5 |
| formoterol | Tocris – 1448 | 10μM | No response | 3 | No response | | 4 | No response | 5 | No response | | 5 |
| guanabenz | Sigma – G110 | 100μM | 23.5 ± 7.4 | 3 | 100μM | 8.5 ± 3.1 | 7 | No response | 5 | No response | | 5 |
| guanfacine | Sigma – G1043 | 100μM | 11.5 ± 2.9 | 3 | 100μM | 2.5 ± 1.7 | 6 | No response | 5 | No response | | 5 |
| idazoxan | Sigma - 16138 | 100μM | No response | 3 | No response | | 5 | No response | 5 | No response | | 5 |
| isoprenaline | Sigma - I5627 | 100μM | No response | 3 | 100μM | 5.1 ± 4.6 | 7 | No response | 5 | No response | | 5 |
| labetolol | Sigma - L1011 | 100μM | 10.3 ± 3.1 | 3 | -5.62 ± 0.04 | 25.9 ± 3.4 | 5 | No response | 5 | No response | | 5 |
| lisuride | Tocris - 4052 | 10μM | No response | 3 | -6.47 ± 0.16 | 41.0 ± 6.4 | 6 | No response | 5 | 10μM | 85.2 ± 5.0 | 5 |
| medetomidine | Tocris - 5160 | 100μM | 11.7 ± 2.4 | 3 | 100μM | 7.9 ± 3.8 | 7 | No response | 5 | 100μM | 88.4 ± 3.2 | 5 |
| metaraminol | Sigma – M4778 | 100μM | No response | 4 | No response | | 5 | No response | 5 | No response | | 5 |
| methoxamine | Sigma – M6524 | 100μM | No response | 3 | No response | | 4 | No response | 5 | No response | | 5 |
| methyldopa | Tocris – 0584 | 10μM | No response | 3 | No response | | 4 | ND | | ND | | |
| α-methylnorepinephrine | Sigma – SML0675 | 100μM | No response | 3 | No response | | 4 | No response | 5 | No response | | 5 |
| midodrine | Sigma – M8277 | 100μM | No response | 3 | No response | | 4 | ND | | ND | | |
| moxonidine | Sellakchem – S2066 | 100μM | No response | 3 | 100μM | 3.1 ± 1.9 | 5 | ND | | ND | | |
| 2-MPMDQ | Tocris - 0661 | 10μM | No response | 3 | No response | | 5 | No response | 5 | No response | | 5 |
| 3-MPPI | Tocris – 0581 | 10μM | No response | 3 | No response | | 5 | No response | 5 | No response | | 5 |
| naphazoline | Sigma – 70170 | 100μM | No response | 3 | 100μM | 9.2 ± 3.3 | 7 | No response | 5 | No response | | 5 |
| noradrenaline | Sigma – A0937 | 100μM | No response | 3 | No response | | 4 | No response | 5 | No response | | 5 |
| octopamine | Tocris – 2242 | 100μM | No response | 4 | 100μM | 2.9 ± 1.8 | 6 | No response | 5 | No response | | 5 |
| oxymetazoline | Tocris - 1142 | 100μM | 3.1 ± 0.9 | 6 | -7.96 ± 0.16 | 46.7 ± 6.0 | 10 | No response | 5 | *-7.71 ± 0.15 | 18.1 ± 2.2% | 5 |
| Para-amino-clonidine | Sigma – A0779 | 10μM | No response | 3 | No response | | 5 | No response | 5 | No response | | 5 |
| PF3774076 | Sigma – PZ0263 | 10μM | No response | 3 | No response | | 5 | No response | 5 | No response | | 5 |
| 2-PMDQ | Tocris – 0627 | 10μM | No response | 3 | No response | | 7 | No response | 5 | No response | | 5 |
| rilmenidine | Tocris - 0790 | 100μM | No response | 3 | No response | | 4 | ND | | ND | | |
| R-phenylephrine | Tocris - 2838 | 100μM | No response | 3 | No response | | 5 | No response | 5 | No response | | 5 |
| RWJ52353 | Tocris - 3935 | 10μM | No response | 3 | No response | | 4 | ND | | ND | | |
| salbutamol | Sigma – S5013 | 100μM | No response | 4 | 100μM | 3.5 ± 2.0 | 7 | ^#^ND | | ND | | |
| salmeterol | Tocris – 1660 | 10μM | 3.2 ± 1.4 | 4 | No response | | 4 | ^#^ND | | ND | | |
| ST-91 | Tocris – 2638 | 100μM | No response | 3 | 100μM | 5.7 ± 3.1 | 6 | No response | 5 | 100μM | 82.5 ± 1.6 | 5 |
| sunepitrion | Sigma – PZ0279 | 100μM | No response | 3 | 100μM | 4.1 ± 2.7 | 6 | ND | | ND | | |
| synephrine | Sigma – S0752 | 100μM | No response | 3 | No response | | 7 | No response | 5 | No response | | 5 |
| T-CG 1000 | Tocris – 5021 | 10μM | No response | 3 | 100μM | 12.3 ± 3.5 | 6 | ND | | ND | | |
| tetrahydrozoline | Sigma – T4264 | 100μM | No response | 3 | No response | | 4 | No response | 5 | No response | | 5 |
| tizanidine | Sellakchem – S1437 | 100μM | No response | 3 | 100μM | 6.7 ± 3.3 | 5 | No response | 5 | No response | | 5 |
| UK14304 | Tocris – 0425 | 10μM | No response | 3 | No response | | 5 | No response | 5 | No response | | 5 |
| xylazine | Sigma – X1251 | 100μM | 2.3 ± 0.3 | 3 | No response | | 6 | ND | | ND | | |
| xylometazoline | Sigma – X6000 | 100μM | 15.8 ± 2.7 | 5 | -7.05 ± 0.17 | 56.2 ± 5.9 | 10 | No response | 5 | *-7.18 ± 0.20 | 21.0 ± 2.4%* | 5 |
| ziprasidone | Sellakchem – S1444 | 10μM | No response | 3 | No response | | 5 | ND | | ND | | |

ND not determined

*oxymetazoline and xylometazoline cause a decrease in forskolin-stimulated cAMP accumulation. The data give are log IC_50_ and % inhibition of forskolin-stimulated cAMP as both compounds caused a decrease in cAMP accumulation (as in supplementary figure 1).

^#^not determined here but no cAMP response was seen in untransfected cells in Baker 2010
